# Socio-economic and Climate Factors Associated with Dengue Fever Spatial Heterogeneity: A Worked Example in New Caledonia

**DOI:** 10.1371/journal.pntd.0004211

**Published:** 2015-12-01

**Authors:** Magali Teurlai, Christophe Eugène Menkès, Virgil Cavarero, Nicolas Degallier, Elodie Descloux, Jean-Paul Grangeon, Laurent Guillaumot, Thérèse Libourel, Paulo Sergio Lucio, Françoise Mathieu-Daudé, Morgan Mangeas

**Affiliations:** 1 Epidemiology of Infectious Diseases, Institut Pasteur, Noumea, New Caledonia; 2 UMR 228, ESPACE-DEV, Institute for Research and Development (IRD), Noumea, New Caledonia; 3 UMR 182, LOCEAN, Institute for Research and Development (IRD), Noumea, New Caledonia; 4 Météo-France, Noumea, New Caledonia; 5 UMR 182, Laboratoire d’Océanographie et du Climat, Expérimentation et Approches Numériques (LOCEAN), Institute for Research and Development (IRD), Paris, France; 6 Department of Internal Medicine and Infectious Diseases, Territorial Hospital Centre, Noumea, New Caledonia; 7 Health Department, Direction of Health and Social Affairs of New Caledonia, Noumea, New Caledonia; 8 Medical Entomology, Institut Pasteur, Noumea, New Caledonia; 9 UMR 228, ESPACE-DEV, Université de Montpellier II, IRD, Montpellier, France; 10 Centro de Ciências Exatas e da Terra (CCET), Universidade Federal do Rio Grande do Norte (UFRN), Campus Universitário—Lagoa Nova, Brazil; 11 UMR 224 MIVEGEC, Institute for Research and Development (IRD), Noumea, New Caledonia; Australian National University, AUSTRALIA

## Abstract

**Background/Objectives:**

Understanding the factors underlying the spatio-temporal distribution of infectious diseases provides useful information regarding their prevention and control. Dengue fever spatio-temporal patterns result from complex interactions between the virus, the host, and the vector. These interactions can be influenced by environmental conditions. Our objectives were to analyse dengue fever spatial distribution over New Caledonia during epidemic years, to identify some of the main underlying factors, and to predict the spatial evolution of dengue fever under changing climatic conditions, at the 2100 horizon.

**Methods:**

We used principal component analysis and support vector machines to analyse and model the influence of climate and socio-economic variables on the mean spatial distribution of 24,272 dengue cases reported from 1995 to 2012 in thirty-three communes of New Caledonia. We then modelled and estimated the future evolution of dengue incidence rates using a regional downscaling of future climate projections.

**Results:**

The spatial distribution of dengue fever cases is highly heterogeneous. The variables most associated with this observed heterogeneity are the mean temperature, the mean number of people per premise, and the mean percentage of unemployed people, a variable highly correlated with people's way of life. Rainfall does not seem to play an important role in the spatial distribution of dengue cases during epidemics. By the end of the 21st century, if temperature increases by approximately 3°C, mean incidence rates during epidemics could double.

**Conclusion:**

In New Caledonia, a subtropical insular environment, both temperature and socio-economic conditions are influencing the spatial spread of dengue fever. Extension of this study to other countries worldwide should improve the knowledge about climate influence on dengue burden and about the complex interplay between different factors. This study presents a methodology that can be used as a step by step guide to model dengue spatial heterogeneity in other countries.

## Author Summary

Dengue fever is the most important viral arthropod-borne disease worldwide and its geographical expansion during the past decades has been of growing concern for scientists and public health authorities because of its heavy sanitary burden and economic impacts. In the absence of an effective vaccine, control is currently limited to vector-control measures. In this context, understanding the sociologic, entomologic and environmental factors underlying dengue dynamics is essential and can provide public health authorities with sound information about control measures to be implemented. In this study, we analyse socio-economic, climatic and epidemiological data to understand the impact of the studied factors on the spatial distribution of dengue cases during epidemic years in New Caledonia, a French island located in the South Pacific. We identify at risk areas, and find that temperature and people’s way of life are key factors determining the level of viral circulation in New Caledonia. Hence, communication campaigns fostering individual protection measures against mosquito bites could help reduce dengue burden in New Caledonia. Using projections of temperature under different scenarios of climate change, we find that dengue incidence rates during epidemics could double by the end of the century, with areas at low risk of dengue fever being highly affected in the future.

## Introduction

Dengue fever is the most important mosquito-borne viral disease, with an estimated 50 million people being infected each year and 2.5 billion people living in areas at risk of dengue worldwide [1]. The true burden of clinically apparent dengue could be twice as high, and the total burden of dengue fever infections could reach 390 million people when including asymptomatic cases [2]. Whereas only nine countries were affected by dengue epidemics in the 1970's, more than a hundred countries are now reporting dengue outbreaks on a regular basis, making dengue fever the most rapidly spreading mosquito-borne viral disease in the world [1,2]. This rapid global spatial spread over the past 40 years probably results from recent socio-economic changes such as global population growth and uncontrolled urbanisation. Lack of effective mosquito control in endemic areas, increased international air traffic or decay in public health infra-structure in developing countries are also important factors that could explain the rapid regional spread of the disease [3–6]. However, in a given country where there are sufficient numbers of susceptible hosts, these factors need to be associated with suitable climate conditions before dengue fever can establish, since it is transmitted by mosquito species whose life cycle is influenced by temperature, humidity and rainfall [7–9]. Indeed, several studies have pointed out that the current geographic distribution of dengue fever or its vector worldwide could be predicted accurately based on climate variables using either statistical models [10] or deterministic models [11–13]. Other studies have pointed out that climate change could have profound consequences on the epidemiology of dengue fever, because increased temperature and rainfall could facilitate viral transmission and could lead to the geographic expansion of the mosquito species responsible for its transmission [11,14–17].

The complex interplay and relative importance of each factor in the occurrence and spread of dengue fever epidemics might differ from one country to another, depending on the specific climate conditions, cultural and socio-economic environment the virus circulates in [18]. Identifying the factors limiting dengue fever spatial spread at a national level could help understanding the worldwide pattern of dengue disease, could help predicting its future spatial distribution, and could provide national decisions-makers with useful information regarding the appropriate control measures to be implemented.

Most studies trying to identify dengue risk factors spatially were performed at a city scale or a local scale (< 80 km) [19–34]. Among these studies, some have identified risk factors for the presence of *Aedes* mosquito species, such as socio-economic factors [29,30], proximity to specific plantations [28,32], proximity of potential breeding sites [22,28,29,32], or human behaviour [30,32]. Some studies highlighted the importance of human movement [23,26,31,33] or population immunity [34] in shaping the spatial transmission of dengue fever at small spatial scales. High dengue incidence rates have also been found in neighbourhoods with low social income [20,26], difficult access to piped water [21,26], or no implementation of mosquito protection measures [21,27].

Spatial analyses at a country or territorial scale (> 200 km) are scarce. Some of these studies focused on the spatio-temporal dynamics of the disease only [35–37] and proposed hypotheses about the underlying processes, but did not include analyses of potential explicative factors. To our knowledge, there are only five studies to date identifying and quantifying spatial risk factors for dengue at a “national” scale > ~200 km and < ~1000 km. Four studies identified temperature as having a major influence on the spatial distribution of locally acquired dengue cases [38–41], the last one did not assess the role of climate factors [42]. The role of other factors in the spatial distribution of dengue cases varied from one place to another. For example, in Australia (Queensland) [38] and Taiwan [40], rainfall seemed to play a minor role whereas in Brazil, rainfall played a major role [39]. In Taiwan [40] and Argentina [41], urbanization level was a key factor in dengue fever spatial distribution, and in one province of Thailand [42], the main factor identified was the proximity to major urban centres. No association was found with socio-economic covariates in Argentina [41] or Australia [38].

New Caledonia, where the present study takes place, has a unique situation: it is a developed insular territory located in the inter-tropical area of the South Pacific where the access to high quality data and the lack of terrestrial borders with other countries make it a natural laboratory to study dengue dynamics. A gross average of ten imported cases is detected each year by the Public Health authorities. However, large dengue epidemics develop only every three to five years, sometimes causing the circulation of the same serotype during two consecutive years [43]. A recent study analysing the temporal relationship between dengue epidemic occurrence and climate variables at an inter-annual scale showed that the development of an epidemic in New Caledonia needs precise climate conditions relying on both temperature and relative humidity [43].

The objectives of the present study were i) to characterise the spatial distribution of dengue cases in New Caledonia once an epidemic spreads over the territory; ii) to determine which of possible covariates are shaping the observed distribution; iii) to explore the potential spatial distribution of dengue cases under future climate projections.

We present a complete methodology, from data collection, data transformation, variable selection, and application to future climate projections. We address a number of methodological issues such as spatial autocorrelation, correlation between explanatory variables, or potential non-linearity between epidemiological data and explanatory covariates.

## Material and Methods

All analyses and figures were performed using R software version 3.2.0 [44], except S3 Fig.

### Study area

**Fig 1:**
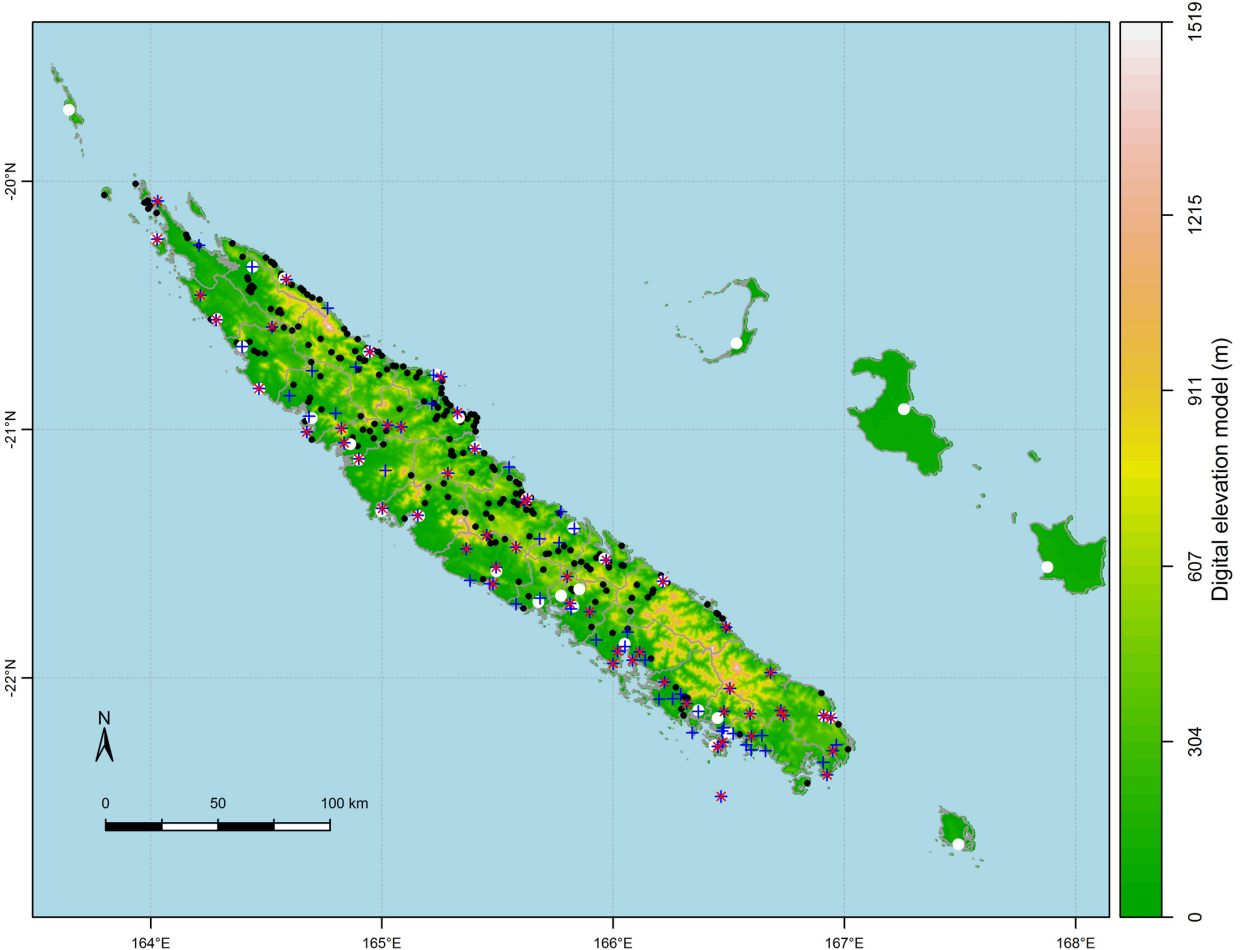
General map of New Caledonia. The map shows the location of towns (white dots), tribes (black dots), and weather stations registering temperature (red crosses) and rainfall (blue crosses) in New Caledonia. The background colour represents the digital elevation model (altitude).

New Caledonia is a French territory, located in the Pacific Ocean 1,500 km East of Australia. It is divided into 33 communes covering 18,576 km^2^. Out of the 245,344 inhabitants (2009), around 58% (147,365 people) live in Noumea, the main city, and its surroundings. The rest of the population is scattered in small towns of about 2,000 people, or live in rural areas, including traditional Melanesian settlements locally called “tribes” (Fig 1). Although the average population density outside Noumea is very low (5.3 inhabitants per km^2^), local densities can be high as people gather in small settlements.

New Caledonia is located at the limit of the tropical zone between latitudes 19° and 23° South. The East coast and the West coast are separated by a mountain range culminating at 1629 m. Climate is heterogeneous: the East coast and the southern tip of the main island get more rain than the West coast, as mountains provide a vertical lift to the warm and humid air brought by trade winds. Average rainfall range from 800 mm/year in some western weather stations to 3200 mm/year in the East. Temperature can drop below 10°C during the cool season on clear nights and sometimes rise above 35°C due to the influence of tropical air masses [45].

From an oversimplified point of view, there are three population groups, having different cultural and social habits: Melanesian people, people of French descent who migrated two hundred years ago, and people from various origins who migrated recently. Although the three groups are spatially partially mixed, Melanesian people live mostly on the East coast, whereas the second group live mostly on the West coast and the third group live mainly in Noumea.

In New Caledonia, dengue represents a major public health problem with large epidemics affecting the territory every three to five years and involving a succession of all four serotypes [43,46–48]. Co-circulation of different dengue virus serotypes (DENV1-4) during major epidemics is rare, and has been observed only once (2009). Before 2003, vector control measures consisted in systematic chemical control of adult mosquitoes covering large areas during the warm season, independently from the occurrence of dengue cases. Since 2003, systematic spreading of adulticide has been stopped, and vector control measures include continuous large communication and prevention campaigns fostering source reduction aimed at all citizens, as well as focal chemical control of adult mosquitoes 100 m around declared cases within 24 h of notification. Public Health infrastructure is reliable, and the surveillance system for dengue fever has been efficient for many years. All people have access to medical care, even though people living in remote areas might have more difficulties to reach local health centres.

### Data

#### Epidemiological data

We studied 24,272 cases of dengue fever reported to the Direction of Sanitary and Social Affairs (DASS-NC) from 1^st^ January 1995 to 30^th^ September 2012, a period over which the spatial location of cases was recorded at the commune level. For each reported case, the date retained to calculate incidence rates was the date of consultation, which can differ from the date of infection by one to two weeks. Cases were recorded on a clinical basis, but more than 71% of declared cases have also been laboratory confirmed either by virus isolation, viral RNA detection (RT-PCR) or NS1 antigen detection (ELISA), or by detection of IgM (ELISA). During the study period, changes in the surveillance system have been homogeneous over the country, making spatial analysis of mean incidence rates possible.

For each of the 33 communes composing New Caledonia, we calculated mean yearly age-standardised incidence rates over epidemic years during the study period, i.e. 1995, 1996, 1998, 2003, 2004, 2008 and 2009. Epidemic years were defined according to the tercile method described in Descloux *et al*. [43]: a year is considered epidemic if the annual incidence rate of this year over the entire territory is in the upper tercile of annual incidence rates calculated between 1971 and 2012 (*i*.*e*. annual incidence rate over the territory > = 18 cases per 10,000 people per year). As the majority of outbreaks display a similar seasonal evolution (beginning in January, epidemic peak between March and May, and end in July) and according to the climate seasonality in New Caledonia, to calculate annual incidence rates, in each commune, we aggregated data annually from 1^st^ September of epidemic year *n-1* to 31^st^ August of epidemic year *n*. We then calculated, in each commune, the average annual incidence rate across epidemic years. Average incidence rates over epidemic years of the study period are a robust indicator of how easily the virus circulates in a given commune during epidemics (i.e. from 1^st^ September of year *n-1* to 31^st^ August of year *n* of the selected years), independently from the inter-annual variability of spatial patterns from one epidemic year to another.

Age-standardisation was necessary as the age structure of the population varies from one commune to another, and age groups are affected differently by dengue epidemics (see S1 Fig). We performed age-standardisation using a direct method [49], which consists in calculating the expected incidence rate in each commune if all communes had the same (arbitrary) age-structure. We chose the World Health Organisation standardised population as the reference population for the arbitrary age-structure [49]. Population numbers and age-structure of each commune used to calculate age-specific incidence rates were interpolated linearly from the 1989, 1996, 2004 and 2009 territorial censuses [50–53].

Annual incidence rates averaged over epidemic years of the study period were artificially low in 4 of the islands surrounding New Caledonia (Mare, Lifou, Ouvéa, Isle of Pines) as the only vector of dengue in New Caledonia, *Aedes aegypti*, was not present over the entire study period [54–56]. We thus decided to limit the analysis to New Caledonia's mainland, comprising 28 communes.

#### Socio-economic covariates

**Fig 2:**
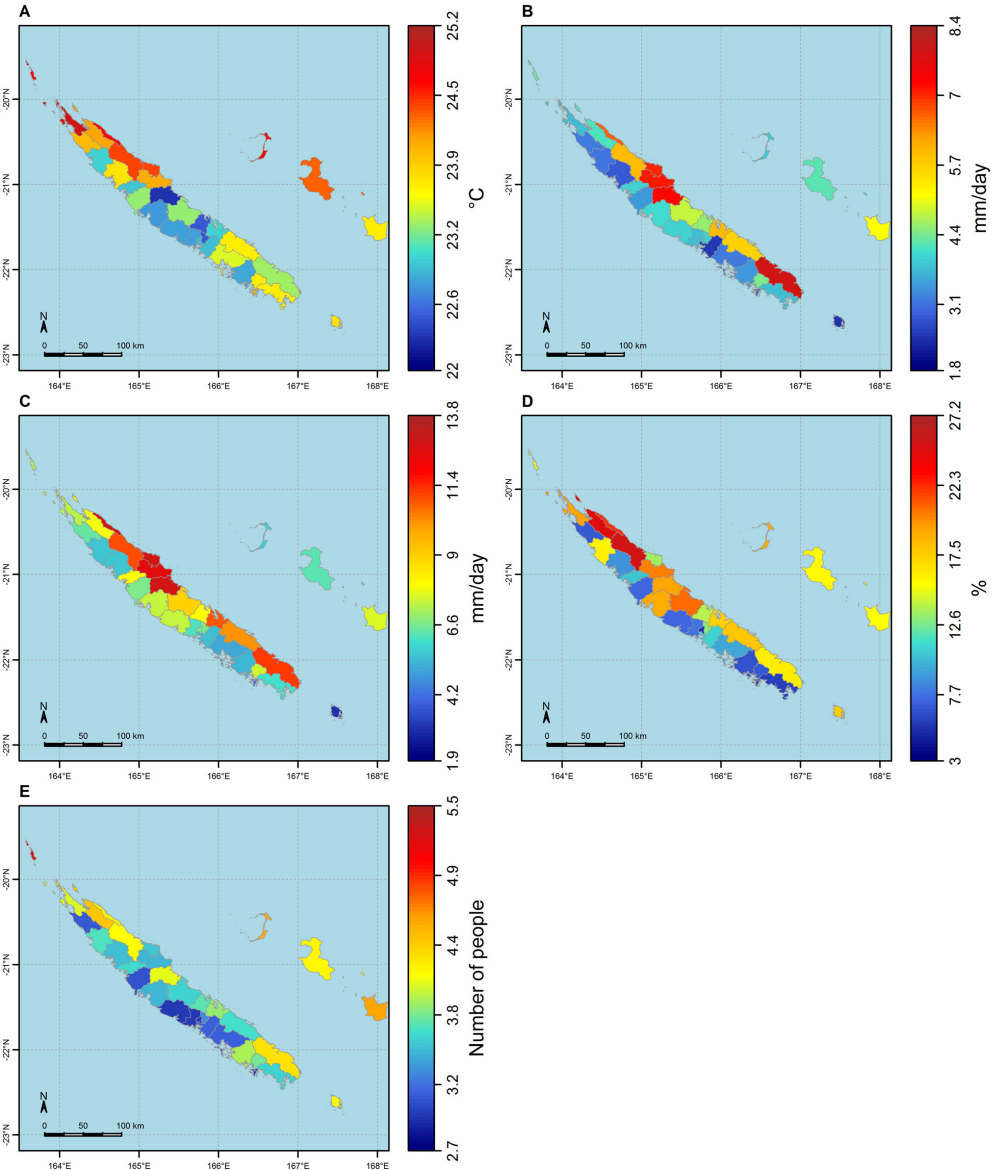
Maps of the 5 explanatory variables selected for modelling dengue incidence rates. **A:** average mean temperature; **B:** average daily rainfall; **C:** average daily rainfall during the wettest quarter; **D:** percentage of unemployed people; **E:** average number of people per premise.

From the 2009 territorial census [53], recorded at the commune level, we selected a total of 51 variables that could potentially influence dengue transmission. These variables can broadly be categorised in sociologic variables reflecting people's activity and financial means, variables linked to human movement within or between communes, variables linked to people's housing, and variables linked to human local density (see Table 1 for a complete list of these variables). Because human density per commune is very low and does not reflect local densities, we decided not to include it in the analysis. Fig 2 shows maps of two of these social variables. In New-Caledonia, the percentage of unemployed people (Fig 2D) varies from 3 to 27% of the population depending on the commune. Unemployment is higher on the East coast than on the West coast, with the fraction of unemployed people being over 10% in most of the eastern communes. The average number of people per household represents local densities (Fig 2E). It varies from 2.9 to 5.3 people per household over the territory, and is highest in the communes in the North-East.

**Table 1:** Correlation between dengue incidence rates and socio-economic or climate variables

Type	Variable	Rho*	p value	Category	Description
**Climate**	**Mean temp**	**0.619**	**0.0004**	**temperature**	**Average mean temperature**
	Coldest quarter	0.617	0.0005	temperature	Average minimum temperature during the coldest quarter
	Coldest month	0.608	0.0006	temperature	Average minimum temperature during the coldest month
	Min temp	0.586	0.0010	temperature	Average minimum temperature
	**Wettest quarter**	**0.563**	**0.0018**	**rainfall**	**Average daily rainfall during the wettest quarter**
	Wettest month	0.559	0.0020	rainfall	Average daily rainfall during the wettest month
	Isothermality	-0.515	0.0051	temperature range	Average of the difference between mean and minimum temperature, divided by mean temperature
	Warmest quarter	0.508	0.0058	temperature	Average minimum temperature during the warmest quarter
	Nb of days max temp 32°C JFM	-0.508	0.0058	temperature range	Average number of days with maximal temperature exceeding 32°C in January, February and March
	Temp range	-0.491	0.0080	temperature range	Average temperature range (difference between maximum and minimum temperature)
	Warmest month	0.483	0.0093	temperature	Average minimum temperature during the warmest month
	**Rainfall**	**0.473**	**0.0109**	**rainfall**	**Average daily rainfall**
	Nb of days rainfall 1 mm Jan	0.419	0.0264	rainfall	Average number of days with daily rainfall exceeding 1mm during January
	Nb of days rainfall 2 mm Jan	0.408	0.0313	rainfall	Average number of days with daily rainfall exceeding 2mm during January
	Driest month	0.296	0.1262	rainfall	Average daily rainfall during the driest month
	Nb of days max temp 30°C JFM	-0.202	0.3015	temperature range	Average number of days with maximal temperature exceeding 30°C in January, February and March
	Driest quarter	0.125	0.5265	rainfall	Average daily rainfall during the driest quarters
	Max temp	-0.062	0.7548	temperature	Average maximum temperature
	Nb of days max temp 28°C JFM	-0.007	0.9700	temperature range	Average number of days with maximal temperature exceeding 28°C in January, February and March
**Altitude**	dem	-0.186	0.3440		Digital elevation model (altitude)
**Socio-economic**	**Activity unemployed**	**0.759**	**< 0.0001**	**Activity**	**Percentage of unemployed people**
	Transport engine	-0.744	< 0.0001	Human movement	Percentage of people using a motorised vehicle to get around
	**Nb people per household**	**0.739**	**< 0.0001**	**Human density**	**Average number of people per premise**
	Transport walk	0.722	< 0.0001	Human movement	Percentage of people getting around by foot
	Activity employed	-0.708	< 0.0001	Activity	Percentage of people working (any occupational activity)
	No car	0.704	< 0.0001	Human movement	Percentage of premises with no car
	Activity other non working	0.648	0.0002	Activity	Percentage of people that are unoccupied but are neither students nor retired
	Proportion tribal population	0.632	0.0003	Activity	Percentage of people living in a tribe
	Spc** agri	0.597	0.0008	Activity	Percentage of people working as farmers
	WC inside	-0.568	0.0016	Housing	Percentage of premises with toilets inside
	Surf < 40 m^2^	0.568	0.0016	Human density	Percentage of premises under 40 m^2^
	House	-0.540	0.0030	Housing	Percentage of premises that are houses
	Live in same commune 04	0.532	0.0036	Human movement	Percentage of people living in the commune before 2004
	Air conditioning	-0.528	0.0039	Housing	Percentage of premises with at least one room with air conditioning
	Live in another commune 04	-0.525	0.0042	Human movement	Percentage of people living in another commune before 2004
	Activity retired	-0.490	0.0081	Activity	Percentage of retired people
	Electricity	-0.488	0.0084	Housing	Percentage of premises with access to public electricity
	Spc** arti	-0.480	0.0097	Activity	Percentage of people working as artisans
	Work other commune	-0.477	0.0103	Human movement	Percentage of people employed in another commune
	Born NC	0.475	0.0106	Activity	Percentage of people born in New Caledonia
	Born France mainland	-0.470	0.0115	Activity	Percentage of people born in France, mainland
	Born FP	-0.466	0.0125	Activity	Percentage of people born in French Polynesia
	Concrete slab	-0.460	0.0139	Housing	Percentage of premises built on a concrete slab
	Surf > 40 & < 120_m^2^	-0.453	0.0154	Human density	Percentage of premises between 40 m^2^ and 120 m^2^
	Surf >120_m^2^	-0.448	0.0169	Human density	Percentage of premises over 120 m^2^
	Born abroad	-0.433	0.0215	Activity	Percentage of people born abroad
	Nb of rooms	-0.428	0.0230	Human density	Average number of bedrooms per premise
	Hut	0.387	0.0421	Housing	Percentage of premises that are huts
	No bikes	0.360	0.0599	Human movement	Percentage of premises with no bicycle nor motorcycle
	Live France mainland 04	-0.349	0.0685	Human movement	Percentage of people living in the mainland of France before 2004
	Individual water	0.345	0.0722	Housing	Percentage of premises using an individual watering place located outside
	Shed	0.330	0.0866	Housing	Percentage of premises that are temporary sheds
	Born WF	-0.305	0.1150	Activity	Percentage of people born in Wallis and Futuna
	Tap water	-0.302	0.1179	Housing	Percentage of premises with access to tap water
	Sector 1	0.262	0.1774	Activity	Percentage of people employed in the primary sector (agriculture…)
	Main home	0.258	0.1849	Human movement	Percentage of premises that are the main home of a household
	Sector 2	-0.244	0.2115	Activity	Percentage of people employed in the secondary sector (industry. . .)
	Spc** executives	-0.233	0.2334	Activity	Percentage of people working as managers or executives
	Spc** labour	-0.206	0.2926	Activity	Percentage of people working as labour workers
	Live abroad 04	-0.170	0.3861	Human movement	Percentage of people living abroad before 2004
	Transport public	0.164	0.4037	Human movement	Percentage of people using public transportation to get around
	Studying	0.148	0.4529	Activity	Percentage of people unoccupied and studying
	Activity undergraduate student	0.148	0.4529	Activity	Percentage of people registered in an undergraduate course
	Collective water	0.132	0.5037	Housing	Percentage of premises using collective watering places
	Sector 3	0.024	0.9015	Activity	Percentage of people employed in the tertiary sector (sales, trade, services. . .)
	Spc** other	0.024	0.9049	Activity	Percentage of people working but not as farmers nor managers nor workmen nor artisans nor employees
	Spc** employee	0.001	0.9958	Activity	Percentage of people working as employees

* Pearson correlation coefficient (Rho) with dengue mean (across epidemic years) annual incidence rates and associated p-value. Variables are sorted by category (socio-economic or climate) and by decreasing order of their absolute Pearson correlation coefficient. Variables selected for the multivariable modelling are in bold

** Spc = Socio-professional category

#### Climate covariates: observed data and upscaling to the communal scale

Observed rainfall, maximal temperature (Max temp) and minimal temperature (Min temp) were provided by Meteo France on a daily basis from 1995 to 2012 in 118 weather stations scattered all over New Caledonia. We first selected data from the same time periods as the epidemiological data (September of epidemic year *n-1* to August of epidemic year *n*). To handle missing data, only time series covering at least a full continuous year were kept resulting in 67 locations for temperature, and 113 locations for rainfall (see Fig 1). For each variable, at each weather station, we then calculated a typical year time-series, 365 days long, by averaging data recorded on the same day and month across the years of the study period (i.e. we averaged data recorded every January 1^st^, every January 2^nd^, etc.). We then calculated the average of these 365 day-long time series. This average is hereafter called “climate index”, thus obtaining one climate index for each climate variable at each weather station.

To model the link between climate and incidence rates by means of regression models, we had to project the punctual station-based climate data to the same spatial level as the epidemiological data (i.e. the commune level). In order to take into account the fact that incidence rates are calculated from individual data, and the fact that the population is distributed heterogeneously within each commune, we assigned every tribe the climate index value of the closest station, assuming all people and all mosquitoes within a tribe to be exposed to the same climate conditions. We then averaged the climate index values over all tribes in the same commune, repeated the process over all towns of the same commune, and then calculated an average of the tribe and town values, weighted by the respective proportion of people living in either place, thus obtaining a mean climate index value per commune (see Fig 2). The distance between a tribe or a town and the closest station ranges from 0 km to 27 km, with a mean of 9 km (see Fig 1). This “projection” process was done for rainfall, minimal and maximal temperature, and for 16 other climate indices built from these three raw variables (see Table 1 for a complete list). The 16 climate indices we built included the key variables most explicative of the inter-annual variability of dengue epidemics in New Caledonia, i.e. the number of days when maximal temperature exceeds 32°C during January/February/March [43], and the number of days when precipitation exceeds 2 mm in January [57]. They also included variables known to have an influence on biological processes (see all other climate indices in Table 1) [58]. Fig 2 shows maps of three of the climate indices built: the mean temperature (Fig 2A), the average daily rainfall (Fig 2B) and the average daily rainfall during the wettest quarter of the year (Fig 2C). Mean temperature as experienced by people during epidemic years ranges from 22°C to 25°C over the territory. Globally, mean temperature experienced by people is higher in the Northern part of New Caledonia than in its Southern part. People living on the East coast are receiving more rainfall (up to 8 mm/day on average over the epidemic years) than people living on the West coast (barely more than 5 mm/day on average over the epidemic years). On the East coast, there are two “hotspots” of heavy rainfall: communes of Touho, Poindimié and Ponérihouen, in the middle-North of the East coast, and Yaté in the South. All observed climate variables and those built from these observed variables are referred to as "observed data" in the article.

As we are here focusing on modelling the spatial variability of dengue incidence rates and not their inter-annual variability, we have not included variables linked to the El Niño Southern Oscillation in the analysis.

#### Climate covariates: assessing the trends of future mean temperature in New Caledonia

To obtain maps of mean temperature under different global warming scenarios, we first retrieved historical (1971–2005) and future (2006–2099) projections of mean temperature simulated by ten coupled ocean-atmosphere models from the 5^th^ Phase of the Coupled Model Intercomparison Project–Assessment Report 4 (CMIP5-AR4) experiments [59,60]. The ten selected models are "bcc-csm1-1", "CanESM2", "CCSM4", "CNRM-CM5", "HadGEM2-CC", "inmcm4", "IPSL-CM5A-MR", "IPSL-CM5B-LR", "MPI-ESM-LR", and "NorESM1-M". They were selected based on their capacity to reproduce a reasonable climate in the South Pacific [61]. All GCM used simulate some ENSO-like variability but with a large disparity [61]. Two scenarios of emission (greenhouse gases and aerosols)—referred to as "Representative Concentration Pathways" (RCPs)–were chosen: RCP 8.5 refers to a high emission scenario while RCP 4.5 refers to a midrange mitigation emission scenario. In this article, these data are respectively denoted "historical" (1971–2005), "RCP 4.5" and "RCP 8.5" (2006–2099).

Model simulations are available at different spatial locations depending on the spatial grid used by each model. For each model, we selected time-series from the spatial point closest to Noumea. We first assessed the necessity of performing a statistical downscaling to correct the retrieved time series so they fit the locally observed temperature time series [62], but concluded that correction was not necessary for estimating average increases in mean temperature in New Caledonia. For the periods 2010–2029 and 2080–2099, we calculated the increase of mean temperature averaged over the 20-year periods compared to the 1980–1999 historical data average (see S2 Fig). By comparing this mean increase to the mean increase in time series taken from other close grid points around New Caledonia, we concluded that this mean increase could be considered homogeneous over the whole territory. As all 10 selected models predictions were coherent (see S3 Fig), we averaged the mean increases over all models. By applying this mean increase to the map of current temperature experienced by people during epidemic years (shown in Fig 2A), we obtained temperature maps for the periods 2010–2029 and 2080–2099 for both climate change scenarios. As projection errors are not readily available for each model, to calculate prediction error of the projections of the mean increase in temperature, for each 20-years period and each scenario, we used the standard deviation across all 10 models as a proxy (see S3 Fig to see the inter-model variability of temperature increase).

#### Altitudinal variation and upscaling to the communal scale

To assess the effect of altitudinal variation on dengue incidence rates, we used the official New Caledonia’s digital elevation model, available on a 10 m resolution grid map ([63] and Fig 1). To obtain a map of the mean altitude of human settlements per commune (variable “dem” in Table 1), we applied the same upscaling algorithm as for the climate data: we assigned each tribe and each city the altitude of the pixel the tribe or city was lying in, then we calculated a mean altitude over all tribes belonging to a same commune, a mean altitude over all cities belonging to a same commune, and finally calculated an average of the tribe and city mean altitude weighted by the percentage of people living in either. We thus obtained a map of mean altitude per commune that takes into account only the altitude of the locations where hosts are present, and where dengue transmission can actually occur. Although mountains in New Caledonia can be as high as 1 629 m, in 24 out of the 28 communes included in the study, people live below 100 m of altitude in average. In 3 communes, people live between 100 m and 150 m in average. In only one commune people live at 250 m in average.

### Multivariable modelling of past dengue incidence rates

#### Spatial autocorrelation of the response variable

In order to assess the global spatial autocorrelation (SAC) of average (across epidemic years) yearly dengue incidence rates over the territory to know whether we had to take it into account in the modelling process, we used semi-variograms [64]. We compared the semi-variogram of observed incidence rates to 200 semi-variograms obtained by random permutations (i.e. without replacement) of the spatial locations of each commune centroid. Distances between pairs of commune centroids were calculated along the roads to take into account the potential influence of the central mountainous chain in the spatial spread of the disease. These semi-variograms measure the global auto-correlation over the entire territory. Therefore, they are not able to detect potential localised auto-correlation. We also assessed the SAC of annual incidence rates for each epidemic year, to assess the presence of global diffusion patterns that could be masked by calculating the average annual incidence rates across epidemic years.

#### Pre-selection of explanatory variables

To reduce the number of variables to study, the number of models to build and to facilitate interpretation, we first used principal component analysis (PCA) [65] separately on climate and socio-economic variables to identify clusters of correlated explanatory variables. In each cluster, we selected the variable most correlated with average (across epidemic years) annual dengue incidence rates as representative of all other variables in the cluster. We were then left with a set of three climate variables and two socio-economic variables as inputs for modelling the incidence rates (see Fig 2).

#### Modelling the spatial association between explanatory variables and dengue incidence rates

To overcome several methodological issues such as multi-collinearity between explanatory variables [66,67] or the possible existence of complex non-linear links between the explanatory and the response variables, we chose to use Support Vector Machines (SVM) to model the link between the five selected explanatory variables and mean yearly dengue incidence rates. SVM is a non-parametric supervised learning algorithm that can be used in high dimensional multivariable classification or regression problems [68,69]. It can be used to model non-linear links without any *a priori* knowledge of the shape of the link [70]. The advantages of using such models are that they do not rely on any assumption regarding the distribution of the response variable; hence there is no need for checking the normality or homoscedasticity of residuals. As they are non-parametric, they are also robust to collinearity between explanatory variables and, compared to classic parametric non-linear models, there is no need for data exploration to specify the shape of the link between the explanatory and the response variables. Another advantage of using such non-linear models to begin with instead of classic linear ones is that explanatory variables potentially linked to the response variable in a non-linear way are not wrongly discarded.

To classify the selected explanatory variables according to their importance in shaping dengue spatial distribution, we compared the overall performance of models based on all possible combinations of one, two or three variables. For each possible combination of explanatory variables, we optimised the SVM parameters combination by building several models using a range of different values for the parameters and selecting the best parameters combination by minimising the models root mean squared errors (RMSE), calculated using a 10-fold cross validation procedure [71]. The RMSE is a global model performance criterion calculated as $RMSE=\sqrt{\frac{\sum_{i=1}^{n}{\left({\hat{y}}_{i}-y_{i}\right)}^{2}}{n}}$, where *y*_*i*_ is the observed dengue incidence rate in commune *i*, ${\hat{y}}_{i}$ is the incidence rate in commune *i* as predicted by the model, and *n* is the total number of communes included in the model. The influence of each explanatory variable on the spatial distribution of dengue cases was then assessed by comparing the RMSE of all optimised models: models based on the best explicative variables have the lowest RMSE.

### Specific model for exploring dengue spatial trends in the future under changing climatic conditions

As described below in the results section, temperature is a key factor determining dengue spatial variability over New Caledonia. We thus decided to explore the evolution of dengue average annual incidence rates during epidemics under changing climate conditions (considering all others variables as remaining constant), by applying the best explicative multivariable model with inputs from maps of temperature for the future (see methods/data/climate covariates: assessing the trends of future mean temperature in New Caledonia). Because the use of kernels in non–linear SVM models impairs predictions outside the observed range of explanatory variables, we built a linear approximation of the best SVM model on present observed data. The linear approximation consists of a simple linear model linking the two best explanatory variables to observed dengue age-standardised average (across epidemic years) annual incidence rates as the response variable. Normality and homoscedasticity of residuals were confirmed by the Shapiro-Wilks' test and the Bartlett's test respectively [67]. To evaluate the error in incidence rates predictions due to the inter-GCM variability of mean temperature increase projections, we calculated, for each time-period and each scenario, the average annual incidence rates during epidemics as predicted by each GCM, and then calculated a standard deviation of predicted annual incidence rates across the different models.

## Results

### Spatial distribution of dengue cases

**Fig 3:**
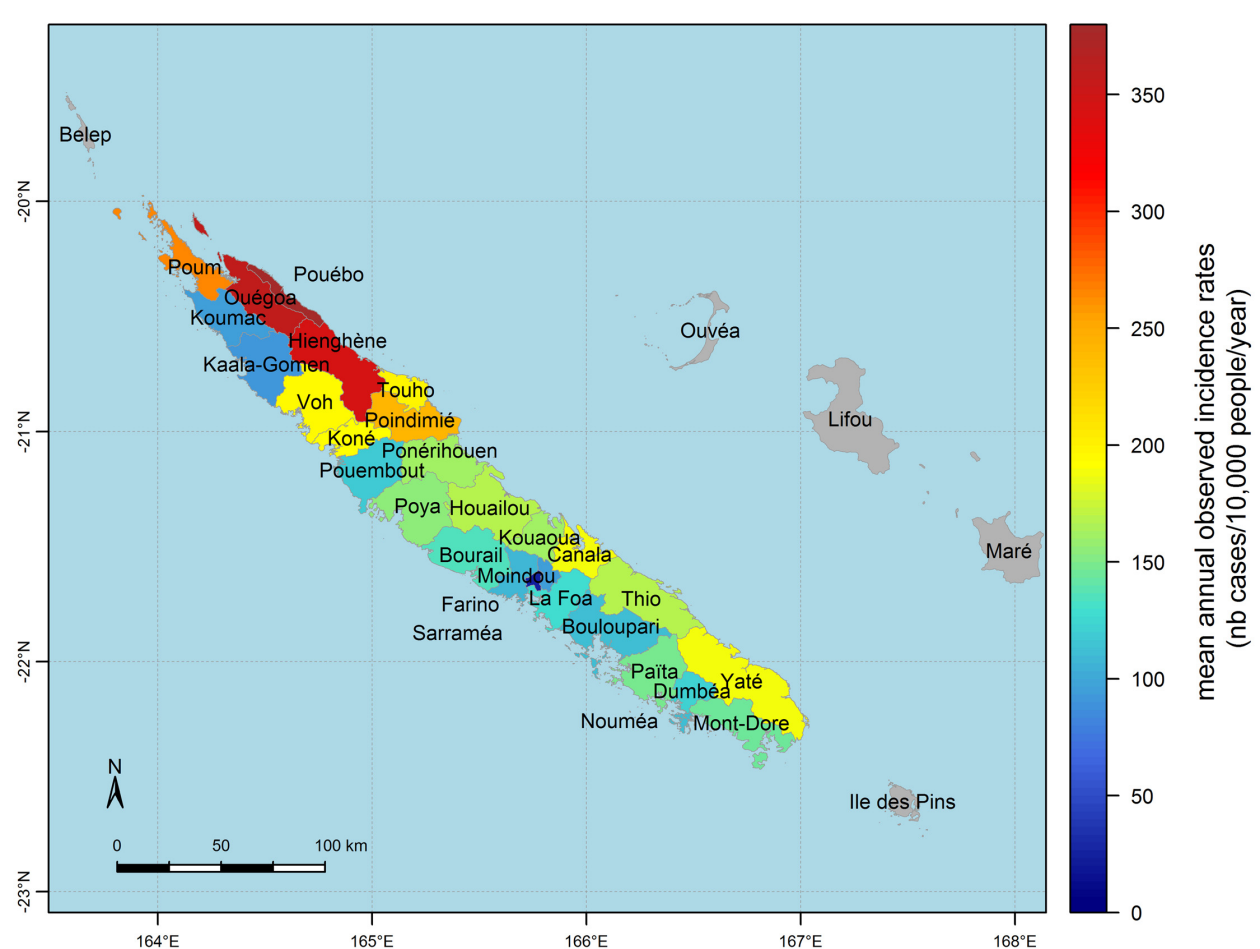
Spatial heterogeneity of dengue annual incidence rates in New Caledonia. Map of annual incidence rates per commune averaged over epidemic years of the 1995–2012 period (years 1995, 1996, 1998, 2003, 2004, 2008, 2009).

Fig 3 shows that once an epidemic spreads over the territory, dengue cases are distributed heterogeneously. Mean annual age-standardised incidence rates across epidemic years range from 22 to 375 cases per 10,000 people per year, with a mean across communes of 168 cases per 10,000 people per year and a standard deviation across communes of 83 cases per 10,000 people per year. On average the East coast is more affected than the West coast. We can also see that the North-eastern corner of New Caledonia is heavily affected, with dengue incidence rates two to three times higher than in the rest of the territory.

By definition, the average across epidemic years of age-standardised annual incidence rates reflects mainly the spatial pattern of severe epidemics, i.e. epidemics of years 1995, 1998, 2003 and 2009. During years 1995, 1998 and 2003, the North-eastern corner was the most affected. During the 2009 epidemic, the most affected communes were Voh and Koné, on the West coast (see Fig 3 for the location of these communes), but the North Eastern corner was still severely affected [72].

### Autocorrelation of the response variable

The semi-variograms of dengue incidence rates did not reveal any significant spatial autocorrelation, whether they were calculated for each epidemic year separately or on the average incidence rates across years. This suggests that the local spread of dengue viruses around a case imported in a commune do not exceed the mean radius of a commune in New Caledonia, e.g. approximately 13 kilometres. Hence, we did not incorporate any spatial structure into the subsequent models.

### Pre-selection of explanatory variables

Table 1 shows Pearson's correlation coefficients (rho) between each explanatory variable and dengue age-standardised annual incidence rates averaged across epidemic years. Dengue is spatially positively correlated with variables related to temperature and precipitation, but is negatively correlated with variables reflecting mean thermal range or extreme thermal conditions (see "Isothermality", "Temp range" or the number of days when maximum temperature exceeds 32°C in January, February and March in Table 1). This suggests that, in a given commune, marked temporal variations of temperature is a factor limiting viral circulation. Based on the linear dependence measure of correlation, dengue is also more strongly associated with temperature than with precipitation. Socio-economic variables are highly spatially correlated to dengue average (across epidemic years) annual incidence rates. Variables reflecting people's way of life (e.g. place of birth), local human density (e.g. mean number of people per household, percentage of premises under 40 m^2^), or human movement are more correlated with dengue average (across epidemic years) annual incidence rates than variables related to the housing type (e.g. premises with inside toilets) (absolute value of rho up to 0.75 for the former and 0.58 for the latter). In particular, the fact that the place where people were born is spatially significantly associated with dengue fever incidence rates (correlation coefficient around 0.5 for people born in New Caledonia and– 0.5 for people born elsewhere) whereas the type of premise is not (absolute correlation coefficient lower than 0.3 for variables describing access to water or electricity) suggests that individual behaviours have a stronger influence on incidence rates than local housing conditions.

**Fig 4:**
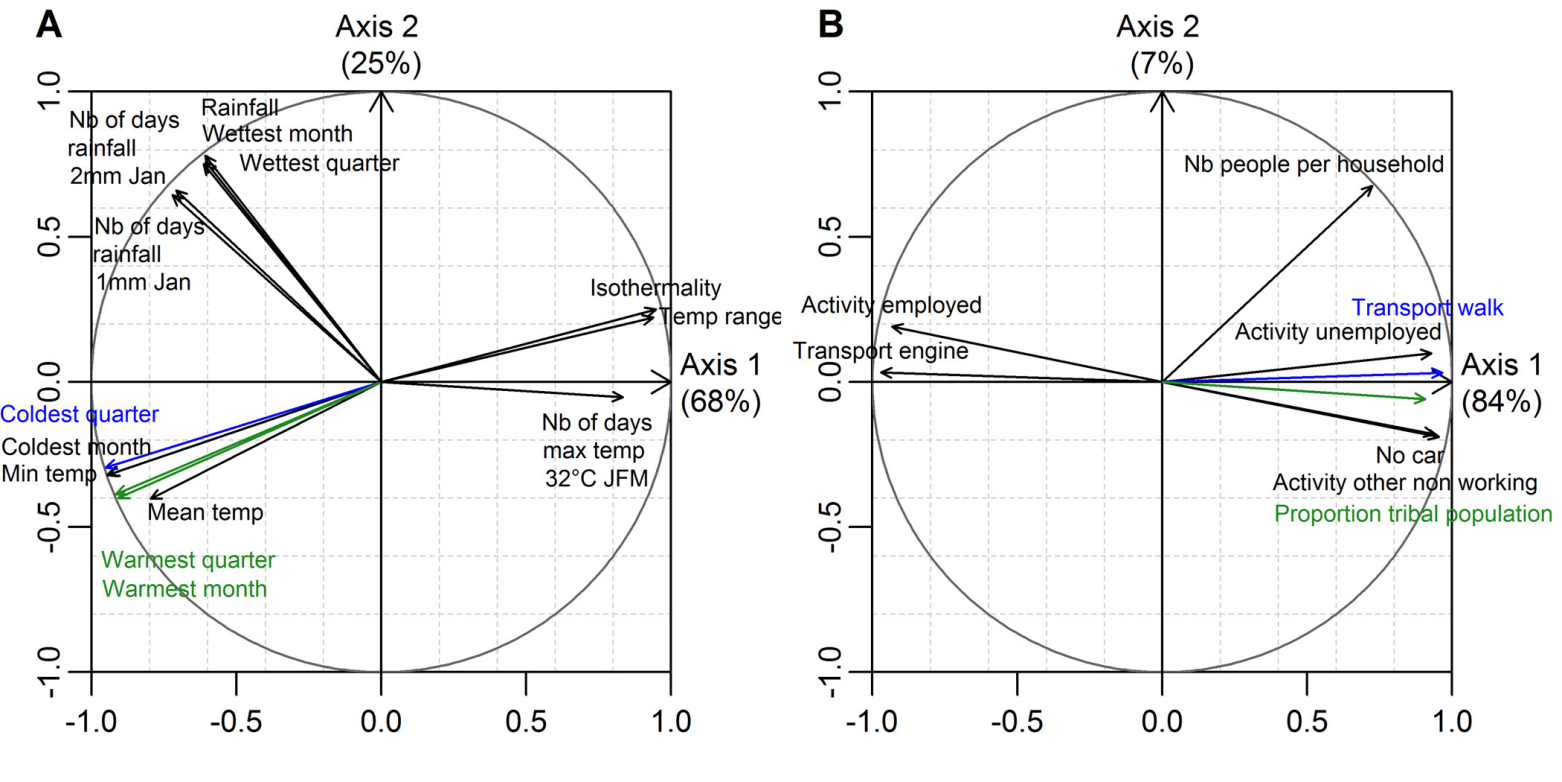
Principal component analysis over the set of climatic variables (A) and socio-economic variables (B). The figure shows the correlation circles of PCA performed on the variables most spatially correlated with dengue average (across epidemic years) annual incidence rates (see methods/multivariable modelling of present dengue incidence rates/spatial autocorrelation of the response variable). Pearson correlation coefficients between variables can be approximated by the angle between the corresponding arrows: 1 for a 0° angle, 0 for a 90° angle, and -1 for a 180° angle.

Fig 4 shows the PCA results. For clarity reasons, we only show the results of PCA performed on the variables most spatially correlated with dengue average (across epidemic years) annual incidence rates, with an absolute Pearson correlation coefficient over 0.6 for socio-economic variables, and over 0.4 for climate variables (these thresholds were selected after verifying that they did not modify the variable pre-selection results).

PCA of climate variables (Fig 4A) shows that in New Caledonia, temperature is the factor accounting for most of the spatial climatic variability among communes. Temperature is highly correlated with the first PCA axis which represents 68% of the total climatic variance. Temperature and rainfall are not spatially correlated at the commune level. In each group of temperature or rainfall variables, the variables most spatially correlated with dengue average (across epidemic years) annual incidence rates were the average mean temperature (Mean temp) and the mean daily rainfall during the wettest quarter of the year (Wettest quarter) (see Table 1). In addition to these two variables, we decided to keep a third variable, the average daily rainfall, for further statistical modelling, as this variable is more easily available in other countries or climate model simulations.

Fig 4B shows that the spatial variability of socio-economic factors mainly reflects the spatial distribution of people with different cultural habits. Communes where a high proportion of inhabitants live in a tribal way, in small premises, with few means of transportation and a high percentage of unemployment are opposed to communes where many people live a western way of life, in permanent buildings, using air conditioning and getting around using cars. Even though the number of people per premise seems to be correlated with the proportion of people living in tribes, we kept this variable as it stands out of the cluster of variables representing the way of life. We thus decided to keep the percentage of unemployed people and the mean number of inhabitants per housing as representative of socio-economic factors for further statistical modelling.

### Modelling the spatial association between explanatory variables and dengue average (across epidemic years) annual incidence rates

Table 2 shows the RMSE of the optimised models built on all possible combinations of one, two or three of the five selected explanatory variables (Mean temperature, daily rainfall averaged over the wettest quarter, average daily rainfall, number of people per household and fraction of unemployed people).

**Table 2:** Univariable and multivariable modelling of dengue average (across epidemic years) annual incidence rates: variable selection according to the RMSE of the SVM models

Variable 1*	Variable 2*	Variable 3*	RMSE**
Activity unemployed	-	-	53
Nb people per household	-	-	68
Mean temp	-	-	69
Wettest quarter	-	-	72
Rainfall	-	-	75
**Mean temp**	**Nb people per household**	**-**	**45**
**Mean temp**	**Activity unemployed**	**-**	**47**
**Activity unemployed**	**Nb people per household**	**-**	**49**
Rainfall	Nb people per household	-	58
Rainfall	Mean temp	-	65
Wettest quarter	Activity unemployed	-	66
Wettest quarter	Mean temp	-	67
Rainfall	Activity unemployed	-	68
Wettest quarter	Nb people per household	-	69
Wettest quarter	Rainfall	-	73
**Mean temp**	**Nb people per household**	**Activity unemployed**	**42**
**Mean temp**	**Nb people per household**	**Rainfall**	**47**
**Mean temp**	**Activity unemployed**	**Rainfall**	**48**
**Mean temp**	**Activity unemployed**	**Wettest quarter**	**49**
**Nb people per household**	**Activity unemployed**	**Rainfall**	**50**
**Nb people per household**	**Mean temp**	**Wettest quarter**	**52**
Nb people per household	Activity unemployed	Wettest quarter	53
Nb people per household	Rainfall	Wettest quarter	63
Mean temp	Rainfall	Wettest quarter	66
Activity unemployed	Rainfall	Wettest quarter	65

* Variables included as explanatory variables for modelling dengue average (across epidemic years) annual incidence rates

** Root mean square error of each model, in number of cases /10,000 people / year. Models are classified first by the number of explanatory variables used, then by increasing RMSE.

Models highlighted in bold perform better than the best univariable model

When looking at univariable non-linear SVM models, the best variable explaining the spatial heterogeneity of dengue average (across epidemic years) annual incidence rates is the percentage of unemployed people per commune. The second most important explanatory variable is the mean temperature. Rainfall is the least explanatory variable of those selected for multivariable regression modelling. Moreover, the RMSE of models based on observed rainfall almost equal the initial standard deviation (across the territory) of dengue average annual incidence rates, which means that rainfall are poor predictors of dengue average annual incidence rates during epidemic years. The relationship between dengue average annual incidence rates and each of the explanatory variables is linear, except for the fraction of unemployed people (S4 Fig). When looking at the spatial structure of dengue average annual incidence rates predicted by SVM models based only on one of the selected variables (S5 Fig), we see that temperature captures mainly the South to North gradient of increasing incidence rates (S5B Fig) whereas socio-economic variables captures the spatial heterogeneity between the West coast and the East coast (S5E and S5F Fig). Temperature seems to have no influence in communes located below 21°S (S5B Fig).

All models based on two explanatory variables and including at least one variable related to rainfall (best RMSE of ~58 cases per 10,000 people per year) performed worse than the best univariable model (RMSE of ~53 cases per 10,000 people per year). This suggests that in New Caledonia, rainfall has little influence on the spatial variability of dengue viral circulation at the commune level. Models combining two explanatory variables (excluding rainfall) performed better than models based on only one variable. The addition of a third explanatory variable did not improve significantly model performances. Hence we focused our attention on models combining two explanatory variables.

**Fig 5:**
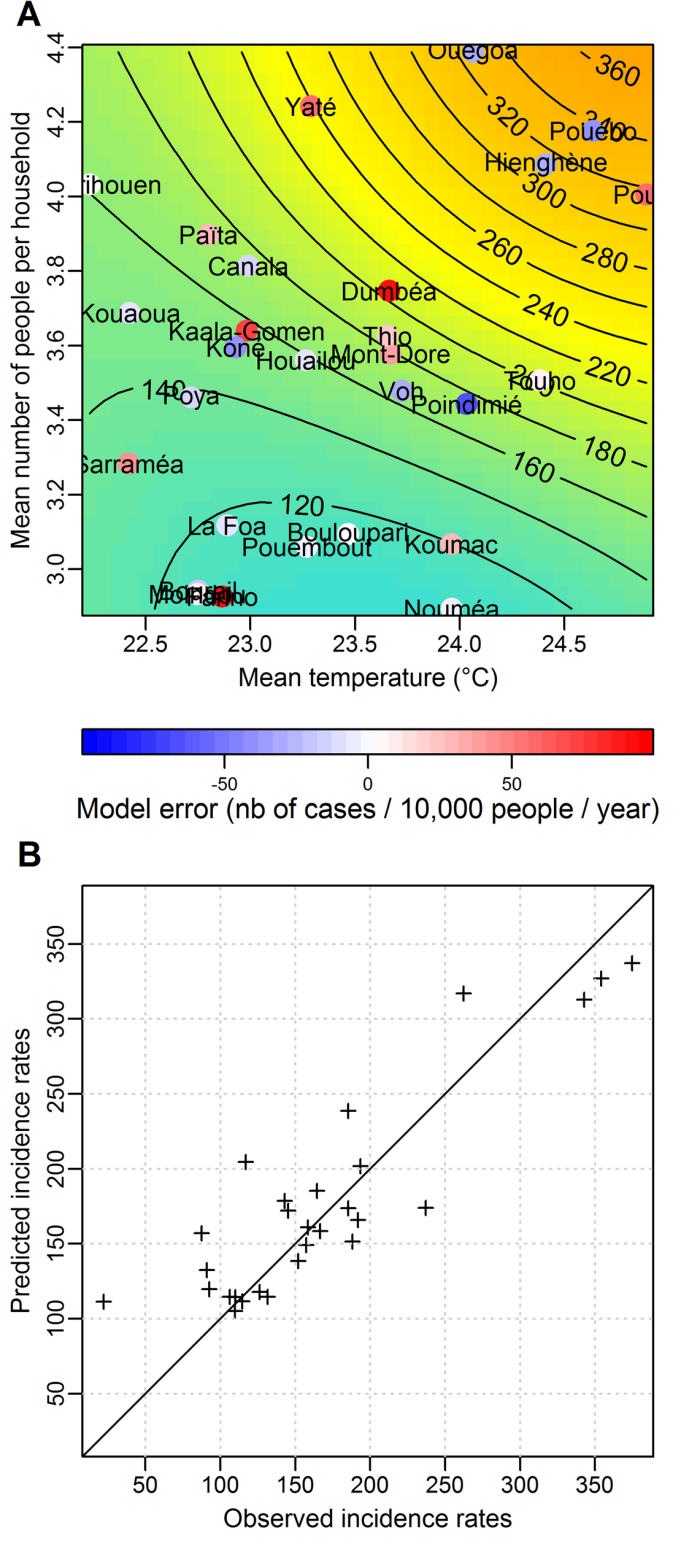
Results of the best multivariable model of the spatial structure of dengue incidence rates. **A:** Predicted mean (across epidemic years) annual incidence rates as a function of the two best explanatory variables (mean temperature and mean number of people per premise). The axes represent the value of the two best explanatory variables. Predicted average annual incidence rates are represented by the colour (blue for low incidence rates to orange for high incidence rates) and by the contour lines giving incidence rates in number of cases per 10,000 people per year. Each commune that has been used to build the model is placed on the graph according to the observed value of the two explanatory variables in the commune. Its position on the graph hence provides the average (across epidemic years) annual incidence rate in the commune as predicted by the model. For each commune, the coloured dot represents the difference between the predicted and the observed incidence rate (model error). **B:** Scatter plot of the predicted and observed average (across epidemic years) annual incidence rates for each of the 28 communes. The RMSE of this model is 45 cases per 10,000 per year.

The best explicative model is a model predicting increasing average annual incidence rates during epidemics in communes where the mean temperature and the mean number of people per premise increase (see Fig 5). The influence of these two variables on the spatial structure of dengue incidence rates is close to linear as shown by almost parallel contour lines on Fig 5A. This model accurately predicts the sharp mean increase in incidence in the three communes of the North East of New Caledonia (Hienghène, Ouégoa and Pouébo). The maximal error of the model is observed for Farino (West coast), which is the only commune where all inhabitants live at an altitude higher than 200 m above sea level.

**Fig 6:**
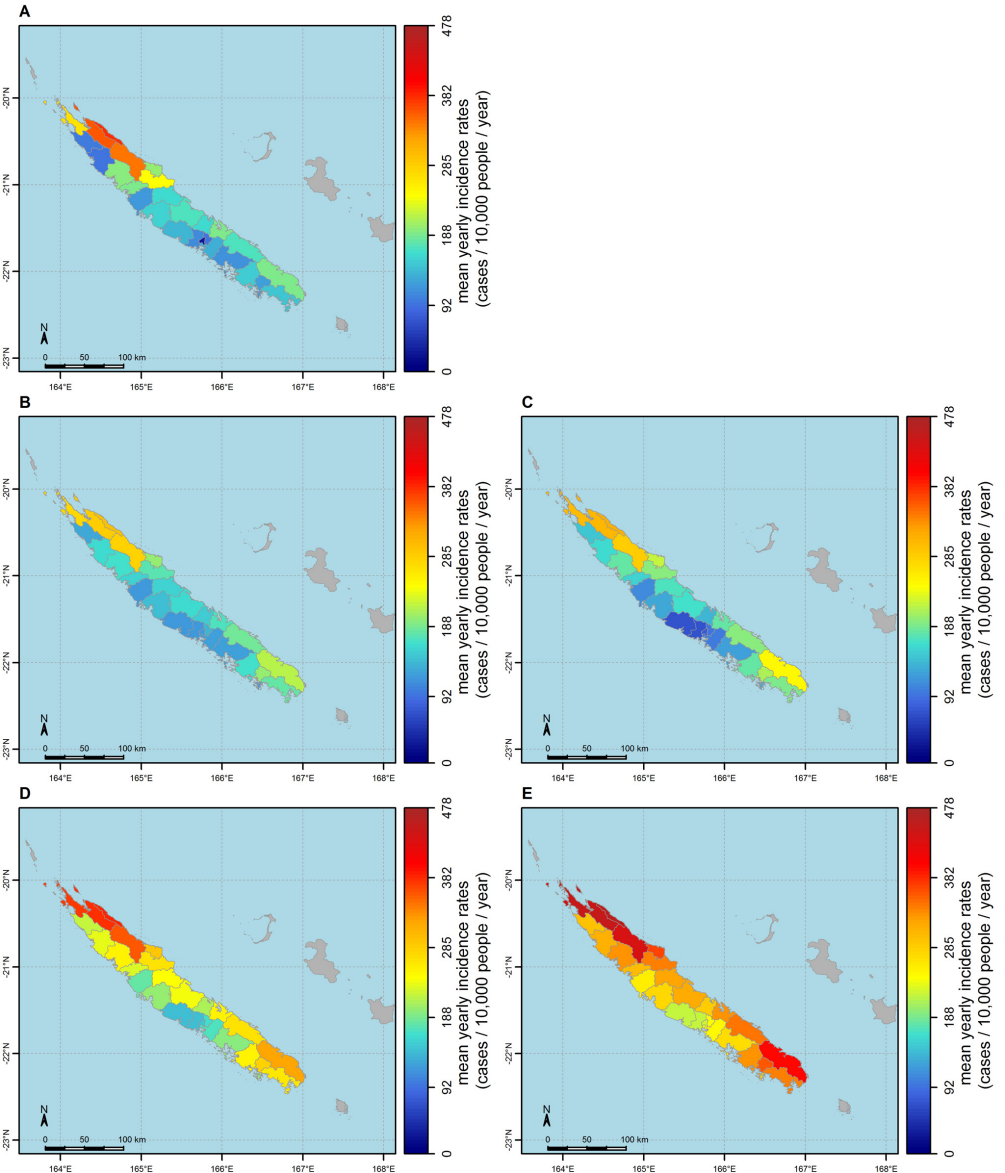
Maps of observed and predicted average annual incidence rates. **A:** map of observed dengue annual incidence rates. **B and C:** maps of dengue annual incidence rates predicted by the SVM model (B) and the linear model (C) based on the mean temperature and the mean number of people per premise (over epidemic years of the study period). **D and E:** Trends of dengue spatial distribution under global warming. Average annual incidence rates during epidemics as projected over the 2080–2099 period under the RCP 4.5 (D) and the RCP 8.5 (E) scenarios.

Fig 6 shows the spatial structure of observed average (across epidemic years) annual incidence rates (Fig 6A) and of average annual incidence rates as predicted by the best SVM model based on two explanatory variables (Fig 6B). This model captures the observed spatial heterogeneity in average annual incidence rates between the East coast and the West coast, as well as the sharp increase in the three communes of the North East.

### Future trends in the spatial distribution of dengue cases under changing climatic conditions

Table 3 shows, for both climate change scenarios, the average increase of mean temperature for the two selected 20-year periods compared to the 1980–1999 historical simulations. All models predict that the mean temperature will increase over time, with projections being more pessimistic for RCP 8.5 simulations. The CMIP5-AR4 inter-model variability in temperature increase is presented in Table 3 and S3 Fig. According to the RCP 8.5 scenario, temperature could increase by more than 3°C by the end of the next century, with a standard deviation across models of only 0.6°C, showing the strong coherency in different model projections.

**Table 3:** Projections of temperature increase and predicted average annual incidence rates during epidemics for three time periods in the future.

Scenario	Period	Projected increase of mean temperature * (°C)	Standard deviation of the projected mean temperature increase** (°C)	Predicted average annual incidence rates during epidemics: territory average across all communes (number of cases / 10,000 people/year)	Standard deviation of the predicted incidence rates**(number of cases / 10,000 people/year)
**-**	1980–1999	-	-	168	-
**RCP 4.5**	2010–2029	0.57	0.14	195	7
	2080–2099	1.53	0.34	241	17
**RCP 8.5**	2010–2029	0.66	0.15	197	8
	2080–2099	3.20	0.60	317	30

* Average of the mean temperature increase predicted by 6 coupled ocean-atmosphere models (see Methods)

** Calculated across the different GCM projections (see S3 Fig for a representation of inter-model variability)

Fig 6 shows a comparison of the average (across epidemic years) annual dengue incidence rates predicted by the SVM model (panel 6B) or the linear model (panel 6C). The SVM model performs slightly better than the linear one: the correlation coefficient between observed and predicted incidence rates are 0.89 (SVM) and 0.85 (linear), and the RMSE are 42 and 43 cases/10,000 people/year respectively for the SVM and the linear model. The low RMSE of the linear model (~43 cases per 10,000 people per year) shows that the linear model based on the two best explanatory variables is suitable. The Shapiro-Wilks and the Bartlett's test confirmed the normality and homoscedasticity of residuals.

Fig 6D and 6E show the potential future spatial distribution of dengue incidence rates during epidemics according to the RCP 4.5 and RCP 8.5 emission scenarios. By the end of the century, dengue incidence rates during epidemic years could reach a maximum of 378 cases per 10,000 people per year in the most affected commune under the RCP 4.5 scenario (Fig 6D), and 454 cases per 10,000 people per year in the most affected commune under the RCP 8.5 scenario (Fig 6E). Under the RCP 8.5 scenario, communes at low risk now might experience a sharp increase in dengue incidence rates during epidemic years from 64 to more than 200 cases per 10,000 people per year. According to RCP 8.5 climate projections, the average (across communes) dengue mean annual incidence rates during epidemic years could raise by 29 cases per 10,000 people per year for the 2010–2029 period, and by 149 cases per 10,000 people per year for the 2080–2099 period, almost doubling dengue burden in New Caledonia by the end of the century (Table 3).

## Discussion

### Association between climate factors and dengue spatial dynamics in New Caledonia

The spatial association found between temperature and dengue incidence rates during epidemics in New Caledonia can be explained by the influence of temperature on the life cycle of the mosquito transmitting the virus in New Caledonia, *Aedes aegypti*. High temperatures increase the productivity of the breeding sites through an acceleration of the metabolism of the mosquito, and a faster development of the micro-organisms the larvae feed on, resulting in a higher vector density even with the same number of breeding sites [7,73–75]. High temperatures also speed up the extrinsic incubation period [7,76,77], with the effect that an increased proportion of females *Ae*. *aegypti* can reach the infectious stage before dying. Finally, warmer temperatures accelerate the mosquito gonotrophic cycle, and make females *Ae*. *aegypti* more aggressive [7,74,78–80], increasing the biting rate and the frequency of potential transmission of viral particles to susceptible hosts. Regarding the effect of increasing temperatures on the mortality of *Ae*. *aegypti* adult mosquitoes, a review of 50 field mark-release-recapture studies has shown that in the field, unless temperatures become extreme (over 35°C or less than 5°C), temperature has little effect on daily mortality rate [81], highlighting the central importance of the length of the extrinsic incubation period in the ability of adult mosquitoes to transmit dengue viruses.

In Noumea, the main city, precise climate variables and important thresholds values have been identified as necessary conditions to trigger an epidemic (e.g. number of days when maximal temperature exceeds 32°C in January/February/March, and number of days when maximal relative humidity exceeds 95% during January [43]). At the scale of the entire territory, we found that the spatial distribution of dengue cases during epidemic years is strongly influenced by the average mean temperature. These results suggest that temperature has a major role in dengue dynamics in an insular territory characterised by climate seasonality. However, we did not find a strong association between the spatial distribution of dengue cases during epidemics and average rainfall or with the number of days when maximal temperature exceeds 32°C. The variables influencing either the triggering of an epidemic [43] or its spatial distribution are not the same. Our findings highlight the complexity of studying and understanding dengue dynamics, the importance of well separating the two epidemiological processes of epidemic triggering in a susceptible population, and its intensity once it has started by clearly defining the modelling target (incidence rates for epidemic intensity, or dummy variables for epidemic triggering), and the importance of well defining the scale of study (temporal evolution, or spatial distribution).

The positive association found between the mean temperature and dengue incidence rates is consistent with the one found in previous studies having analysed the spatial distribution of dengue cases at spatial scale > 200 km [38–42]. In these studies as well as in ours, all regions were located between 10° and 25° of latitude, at the fringe of the tropical area, except Argentina, where the region studied extends to 35° South. In the 10° ˗ 25° latitudinal band, annual mean temperature lies in a range of temperature where the life cycle of the mosquito is very sensitive to temperature changes [7].

Some *Aedes* species, including *Ae*. *aegypti*, are able to breed in very small amounts of water, e.g. snails’ shells. Rainfall can play a role in dengue transmission cycle by filling up potential breeding sites [7], thus influencing the vector density. Rainfall also increases the relative humidity, which extends the mosquitoes’ lifespan and therefore the likelihood of those who had an infectious blood meal to reach the infectious stage. However, our study suggests that in New Caledonia, there is no strong association between rainfall and the spatial distribution of cases during epidemics. A plausible explanation can be the multi-factorial nature of dengue fever, and the relative influence each factor plays on dengue dynamics: despite suitable rainfall conditions, dengue might not circulate well if other factors are limiting dengue viral circulation, such as some human behaviour influencing the contact between vector and host. This aspect has been highlighted very clearly in the United States [27]. Some studies have found that the effect of rainfall on vector density can be modulated by human activities such as water storage practices [82]. However, in New Caledonia, we are not aware of specific practices to store water that could explain the lack of association between rainfall and virus circulation intensity. Another potential explanation could be that in dry areas, breeding sites are filled up by other non-climatic mechanisms, such as automatic irrigation or plant watering.

Worldwide, the spatial association between rainfall and the spatial distribution of dengue cases at a “national” scale (> 200 km) is not as clear as the one for temperature: one spatial study did not find any association between dengue incidence rates and rainfall [40], whereas two others did [38,39]. Other factors influencing dengue transmission (e.g. anthropogenic factors influencing the availability of filled breeding sites) and not included in the different studies might blur the rainfall signal.

It would be interesting to perform the same kind of multi-factorial spatial analysis in areas of epidemic or endemic transmission located closer to the equator, where the mean temperature is higher, to see what climatic factors impact the spatial distribution of cases. This kind of study could help understand better the complex interplay between the different factors (climate, socio-economic, immunologic, viral, entomologic…) associated with dengue fever transmission.

### Association between socio-economic factors and the spatial distribution of dengue cases in New Caledonia

Regarding the link between socio-economic variables and dengue incidence rates during epidemics, a limitation of this study is the absence of historical time series of socio-economic variables. We then had to assume that the data retrieved from the 2009 census is representative of the mean socio-economic spatial pattern over the epidemic years of the 1995–2012 period. As there has been no major historical event leading to population migration in New Caledonia during this time period and as socio-economic variables represent mainly people’s way of life, we think this assumption is realistic.

Our results are consistent with previous studies that have pointed out the importance of socio-economic factors on the spatial distribution of dengue cases, whatever the spatial scale studied: national (>200 km) [39–42] or local (<10 km) [20,26,29,83]. The spatial association between the percentage of unemployed people and dengue in New Caledonia cannot be interpreted in terms of lack of economic activity only, as shown by the PCA on socio-economic factors. This variable we selected as input for the models is highly correlated with other variables reflecting the way of life, socio-economic and cultural differences existing in New Caledonia, which are in turn highly correlated to housing type. Therefore, at this spatial scale in New Caledonia, it is difficult to statistically differentiate the role played by human behaviour, human activity or housing type in dengue fever transmission. However, those three factors influence the contact rate between viraemic patients or susceptible hosts on one hand, and mosquitoes on the other hand. This highlights the importance of limiting the contact between humans and vectors and should lead local authorities to strengthen communication campaigns about personal protection measures towards populations at risk.

Regarding the spatial association found between the fraction of unemployed people (i.e. people’s way of life) and dengue incidence rates during epidemics in New Caledonia, it is interesting to point out that on the East coast, a larger fraction of inhabitants are Melanesian people living in tribes, whereas on the West coast, the majority of people are people from French descent having a western way of life. It would be interesting to perform sociologic studies to precisely identify which human behaviour leads to an increased risk of catching dengue fever. Such information would be useful to define communication messages towards at risk populations.

The spatial association between the number of people per household and dengue incidence rates can be explained by the short flight range of *Ae*. *aegypti* mosquitoes. These mosquitoes are often captured in the very house where they emerged or in the neighbouring houses, flying an average of 40 to 80 m during their life [84–87]. Hence, dengue outbreaks involving *Ae*. *aegypti* as the main vector are known to be highly spatially focal, with dengue cases usually clustering within 200 m to 800 m of each other [23,33,34,88–94]. Our results suggest that, in New Caledonia, dengue cases probably cluster within houses. Sick people should protect themselves until they are no longer vireamic to avoid human to mosquito transmission, and people living around a case should protect themselves to avoid getting infected while infectious mosquitoes are still active in the neighbourhood. Taking such individual actions could reduce the intensity of dengue transmission and reduce dengue burden over the territory. This message could be strengthened in the recommendations given by the authorities.

### Influence of global warming on future dengue incidence rates in New Caledonia

The results about climate change must be interpreted keeping in mind that they represent a climate risk only, and that the spatial association between dengue incidence rates during epidemics and temperature might change over time depending on socio-demographic changes, or changes in dengue control strategy. Assuming all other factors remain constant in time, our results suggest that Public Health authorities can expect the dengue burden to raise significantly during the next century over the territory, and can expect the dengue spatial range to increase. As the GCM projections are spatially homogeneous over the territory, and as the model used to predict dengue incidence rates in the future is linear and is based on only one climate variable, the predicted absolute increase in dengue incidence rates is currently the same for all communes. This highlights the need for spatially downscaling GCM projections to gain a better understanding of the impact of climate change in the future.

Communes that are already severely affected by dengue epidemics will have to prepare to face higher burden of dengue fever. For communes that are at low risk now, we can see that in the future they might be affected as severely as communes at high risk now. These communes might not be prepared now to face severe epidemics of dengue fever, and they will probably need support for adaptation.

As said earlier, the positive association found between temperature and dengue incidence rates during epidemics for mean temperatures ranging from 22°C to 25°C can be explained by the effect of temperature on the mosquito life cycle and duration of extrinsic incubation period. Here, by applying a statistical model built using current observed temperature to future projections, we make the assumption that the biological effect of temperature on the mosquito life cycle and on the extrinsic incubation will remain the same under the range of temperature that might be observed in the future. For most parameters influencing transmission, this assumption is reasonable. For example, we know that in Thaïland, for DENV-2, the extrinsic incubation period is reduced from 15 days at 30°C to 7 days at 32–35°C [77], which is in support of increasing temperatures inducing an increase in dengue incidence rates under future climate. However, because we used a statistical model, we were not able to incorporate the known negative effect that an increase in temperature might have on dengue transmission when temperature reaches extremes. For example, a review of fifty mark-release-recapture studies has shown that the survival and longevity of *Ae*. *Aegypti* mosquitoes is highly reduced when temperatures exceed a threshold, which might be around 35°C [81]. It would be interesting to develop models that are able to integrate these negative effects in the future in order to gain a better understanding of the effects of climate change on dengue transmission.

### Type of epidemiological data used

Here we used data collected routinely by the Direction of Sanitary and Social Affairs. As any surveillance system, it is highly probable that not all dengue cases have been recorded. However, in New Caledonia, the data is of high quality, and the spatial standardisation of the surveillance system (i.e. all the actions taken to be able to compare data collected by different people, at different places [95]) is good, which means that the proportion of cases that are not recorded by the surveillance system are probably comparable from one commune to another. Hence, maps of incidence rates calculated from routinely collected data can be used to study the spatial variability of true incidence rates.

To calculate mean incidence rates, we have used the consultation date of cases. Consultation can occur 1 to 5 days after the onset of symptoms, and the incubation period lasts 4 to7 days on average [3], which means that the consultation date can differ from one to two weeks from the date of infection. This loss of temporal precision is not important here to calculate maps of incidence rates, as for each commune, we have averaged incidence rates across many years.

As in any epidemiological spatial study using routinely collected surveillance data, it is possible that some spatial bias has been introduced due to the fact that the spatial data recorded is the commune where people live, which can sometimes differ from the commune where they got infected.

Another limitation of using routinely collected data is that only clinically apparent cases are recorded, dismissing clinically inapparent cases, whose proportion can vary in time and space [96,97]. A seroprevalence survey is currently undertaken by the Public health authorities. It will be interesting to compare the spatial distribution of seroprevalence to the spatial distribution of average incidence.

### Methodological issues

The analysis of the spatial pattern of infectious diseases, in relation with environmental or socio-economic factors raises a number of methodological issues, such as the presence of spatial auto-correlation, the spatial scale of aggregation of the data, the existence of possible non-linear links between the response and the explanatory variables, or the presence of multi-collinearity between the response variables. Most issues have already been addressed in the past, and solutions already exist to handle them. For example, the reviews by Dormann *et al*. deal with multi-collinearity [66] or spatial-autocorrelation [98]. The main issue we have been confronted with in our study was the spatial upscaling of meteorological data observed at precise locations to the same spatial level of aggregation as the epidemiological data. In existing spatial studies of dengue fever at a national scale, authors have geo-spatially interpolated climate variables on regular grids using kriging methods, and have averaged gridded values over a given administrative division [38–41]. This approach has two drawbacks in New Caledonia. Simple kriging models do not take into account the potential elevation between two given points, leading to biased estimates of temperature in mountainous regions. Moreover, the traditional approach used in climatology, which consist in aggregating temperature over grid points taken uniformly over the whole aggregative area makes the implicit assumption that people at risk are distributed homogeneously over the aggregative area. This is particularly problematic in New Caledonia where large areas are not inhabited. In our approach, as epidemiological data are collected at the individual level, we tried to estimate the climate conditions for each individual (and therefore for the mosquitoes surrounding each individual). However, the algorithm used introduces some noise, due to the fact that the weather stations are sometimes kilometres away from some towns or tribes. High spatial resolution climate data obtained from high resolution modelling of atmospheric conditions could be used, but some noise will be introduced by the modelling error compared to the observed data. This issue needs further attention in the future to increase the quality of spatial epidemiological and environmental studies.

### Factors not taken into account and perspectives

Some factors that could influence the spatial distribution of dengue cases during epidemics have not been taken into account in this study: the location of the first cases introduced each year, the spatial variability in population immunity, viral factors such as the serotype circulating, or factors associated to the mosquito such as the spatial variability in vector competence, or dengue vector control measures. We decided not to include the serotype, as we performed the analysis on averages over several years, and as, except in 2009, there was no co-circulation of different serotypes over the territory. Therefore, spatial differences in the level of viral circulation cannot be associated with genetic differences between serotypes. A territorial seroprevalence survey to assess population immunity has been implemented recently in New Caledonia, but data are not available yet. It could be interesting to include environmental variables derived from GIS data or remote-sensing in this kind of study. For example, GIS data about built areas could be used to create indicators of the proximity of houses to reflect the fragmentation of the *Ae*. *aegypti* habitat per commune, given that fragmentation of this habitat could potentially slow down viral circulation. The amount of vector control effort implemented in each commune is heterogeneous on the territory, as this activity falls within the commune’s authority, and each commune is free to implement or not the territorial guidelines. The vector control effort in each commune is thus difficult to quantify, and data were not available yet at the time of analysis. As soon as these data will be collected by local authorities, they could be incorporated in the modelling process to assess the efficiency of vector control measures.

The study we present here is about the spatial heterogeneity of dengue incidence rates across epidemic years, independently of the inter-annual variability of dengue incidence rates from one epidemic year to another. The mean spatial pattern studied is very robust to changes in the definition of an epidemic year, as severe epidemics will always be considered in the calculation of the mean, whatever the threshold used to distinguish epidemic from non-epidemic years. It would be interesting to know whether the spatial association found here between the severity of dengue epidemics, temperature, local people’s density and people’s way of life is consistent through time or not, and to identify the factors associated to the temporal variability of spatial patterns.

Two other viruses transmitted by *Ae*. *aegypti* like dengue virus caused outbreaks recently in New Caledonia. Chikungunya virus has been introduced on four occasions since 2011 but in each case, the outbreaks were limited to a few cases in Noumea and surroundings. Conversely, Zika virus caused large epidemics over the territory in 2014 and 2015, with more than 1,500 confirmed cases and more than 11,000 estimated cases. Although these viruses are transmitted by the same mosquito as dengue fever, no sufficient data are available to know if the socio-economic and climatic factors driving epidemics are the same. It is likely that local vector competence and population immunity represent major limiting factors. Although dengue has caused major outbreaks in NC in 2013, chikungunya viruses have only caused a limited number of cases for reasons that remain unexplained today and despite the competence of local *Ae*. *aegypti* for chikungunya virus transmission [99]. It is likely that climatic factors and interactions between viruses circulating together between human-hosts and mosquito-vectors influence the epidemiology of arboviruses in New Caledonia. A comparative analysis of the spatio-temporal distribution of these three arboviruses in an insular territory accommodating only *Ae*. *aegypti* represents an important issue to understand and predict outbreaks.

## Supporting Information

S1 Fig Mean dengue annual incidence rates according to age classes. Incidence rates are averaged over epidemic years between 1995 and 2012 and are shown in number of cases per 10,000 people per year.
(TIF)

S2 Fig Increase of mean temperature in Noumea as projected by ten coupled atmosphere-ocean models at the 2100 horizon. The yellow-green curve (1971–2005) represents the observed mean temperature in Noumea. The other curves represent the average of the annual time series simulated by the ten models over the historical period (1971–2005, red curve), and the 2006–2099 period under RCP 4.5 (blue curve) and RCP 8.5 (green curve) scenarios. Horizontal segments represent the average of the time series over the given periods (1980–1999, 2010–2029 and 2080–2099). Arrows represent the average increase in mean temperature for each time period (see Table 3). Background shadings represent the annual range of mean temperature simulations over the ten models.
(TIF)

S3 Fig Distribution of ten coupled ocean-atmosphere model projections for the increase in mean temperature in Noumea. The boxplot (over the ten GCM selected) of the average increase in mean temperature is given for two climate change scenarios (RCP 4.5 and RCP 8.5) and two time periods relative to the historical series of temperature (1980–1999).
(TIFF)

S4 Fig Relationship between dengue annual incidence rates and the 5 selected explanatory variables. For each selected explanatory variable (A to E), mean annual incidence rates observed in 28 communes of New Caledonia (crosses), and mean annual incidence rates predicted by the univariable SVM model based on the corresponding explanatory variable (dots). The curve represents the univariable SVM model predictions over the whole observed range of the explanatory variable (non-linear regression curve). RMSE of each model are 53 (A), 68 (B), 69 (C), 72 (D), 75 (E), as presented in Table 2.
(TIFF)

S5 Fig Spatial influence between the selected explanatory variables and dengue incidence rates. **A:** map of the observed mean dengue annual incidence rates. **B to E**: maps of mean annual incidence rates predicted by univariable SVM models based only on one of the 5 selected variables: mean temperature (B), average daily rainfall (C), average daily rainfall during the wettest quarter (D), percentage of unemployed people (E) and mean number of people per household (F).
(TIF)

## References

[1] WHO. Dengue Guidelines for Diagnosis, Treatment, Prevention, and Control. [Internet]. Geneva: TDR: World Health Organization; 2009 Available: http://site.ebrary.com/id/10363988 23762963

[2] Bhatt S, Gething PW, Brady OJ, Messina JP, Farlow AW, Moyes CL, et al The Global Distribution and Burden of Dengue. Nature. 2013;496: 504–507. 10.1038/nature12060 23563266 PMC3651993

[3] Gubler DJ. Dengue and Dengue Hemorrhagic Fever. Clin Microbiol Rev. 1998;11: 480–496. 9665979 10.1128/cmr.11.3.480 PMC88892

[4] Guzman MG, Halstead SB, Artsob H, Buchy P, Farrar J, Gubler DJ, et al Dengue: a Continuing Global Threat. Nat Rev Microbiol. 2010;8: S7–S16. 10.1038/nrmicro2460 21079655 PMC4333201

[5] Kyle JL, Harris E. Global Spread and Persistence of Dengue. Annu Rev Microbiol. 2008;62: 71–92. 10.1146/annurev.micro.62.081307.163005 18429680

[6] Monath TP. Dengue: the Risk to Developed and Developing Countries. Proc Natl Acad Sci. 1994;91: 2395–2400. 8146129 10.1073/pnas.91.7.2395 PMC43378

[7] Focks DA, Haile DG, Daniels E, Mount GA. Dynamic Life Table Model for *Aedes aegypti* (Diptera: Culicidae): Analysis of the Literature and Model Development. J Med Entomol. 1993;30(6): 1003–1017. 8271242 10.1093/jmedent/30.6.1003

[8] Focks DA, Daniels E, Haile DG, Keesling JE. A Simulation Model of the Epidemiology of Urban Dengue Fever: Literature Analysis, Model Development, Preliminary Validation, and Samples of Simulation Results. Am J Trop Med Hyg. 1995;53 (5): 489–506. 7485707 10.4269/ajtmh.1995.53.489

[9] Morin CW, Comrie AC, Ernst K. Climate and Dengue Transmission: Evidence and Implications. Environ Health Perspect. 2013;121: 1264–1272. 10.1289/ehp.1306556 24058050 PMC3855512

[10] Hales S, De Wet N, Maindonald J, Woodward A. Potential Effect of Population and Climate Changes on Global Distribution of Dengue Fever: an Empirical Model. The Lancet. 2002;360: 830–834. 10.1016/S0140-6736(02)09964-6 12243917

[11] Jetten TH, Focks DA. Potential Changes in the Distribution of Dengue Transmission under Climate Warming. Am J Trop Med Hyg. 1997;57 (3): 285–297. 9311638 10.4269/ajtmh.1997.57.285

[12] Degallier N, Favier C, Menkes CE, Lengaigne M, Ramalho WM, Souza R, et al Toward an Early Warning System for Dengue Prevention: Modeling Climate Impact on Dengue Transmission. Clim Change. 2010;98: 581–592.

[13] Hopp MJ, Foley JA. Worldwide Fluctuations in Dengue Fever Cases Related to Climate Variability. Clim Res. 2003;25: 85–94.

[14] McMichael AJ, Woodruff RE, Hales S. Climate Change and Human Health: Present and Future Risks. The Lancet. 2006;367: 859–869. 10.1016/S0140-6736(06)68079-3 16530580

[15] Githeko AK, Lindsay SW, Confalonieri UE, Patz JA. Climate Change and Vector-borne Diseases: a Regional Analysis. Bull World Health Organ. 2000;78: 1136–1147. 11019462 PMC2560843

[16] Naish S, Dale P, Mackenzie JS, McBride J, Mengersen K, Tong S. Climate Change and Dengue: a Critical and Systematic Review of Quantitative Modelling Approaches. BMC Infect Dis. 2014;14: 167 10.1186/1471-2334-14-167 24669859 PMC3986908

[17] Patz JA, Martens WJ, Focks DA, Jetten TH. Dengue Fever Epidemic Potential as Projected by General Circulation Models of Global Climate Change. Environ Health Perspect. 1998;106: 147–153. 9452414 10.1289/ehp.98106147 PMC1533051

[18] Reiter P. Climate Change and Mosquito-borne Disease. Environ Health Perspect. 2001;109: 141–161. 11250812 10.1289/ehp.01109s1141 PMC1240549

[19] Aziz S, Ngui R, Lim Y a. L, Sholehah I, Nur Farhana J, Azizan AS, et al Spatial Pattern of 2009 Dengue Distribution in Kuala Lumpur Using GIS Application. Trop Biomed. 2012;29: 113–120. 22543611

[20] Costa JV, Donalisio MR, Silveira LV de A. Spatial Distribution of Dengue Incidence and Socio-environmental Conditions in Campinas, São Paulo State, Brazil, 2007. Cad Saúde Pública. 2013;29: 1522–1532. 10.1590/0102-311X00110912 24005918

[21] Bohra A, Andrianasolo H. Application of GIS in Modeling Dengue Risk Based on Sociocultural Data: Case of Jalore, Rajasthan, India. 2001; Available: http://repository.searo.who.int/handle/123456789/15847

[22] Estallo EL, Más G, Vergara-Cid C, Lanfri MA, Ludueña-Almeida F, Scavuzzo CM, et al Spatial Patterns of High *Aedes aegypti* Oviposition Activity in Northwestern Argentina. Noor AM, editor. PLoS ONE. 2013;8: e54167 10.1371/journal.pone.0054167 23349813 PMC3547876

[23] Honório NA, Nogueira RMR, Codeço CT, Carvalho MS, Cruz OG, de Avelar Figueiredo Mafra Magalhães M, et al Spatial Evaluation and Modeling of Dengue Seroprevalence and Vector Density in Rio de Janeiro, Brazil. Gubler D, editor. PLoS Negl Trop Dis. 2009;3: e545 10.1371/journal.pntd.0000545 19901983 PMC2768822

[24] Khormi HM, Kumar L, Elzahrany RA. Modeling Spatio-temporal Risk Changes in the Incidence of Dengue Fever in Saudi Arabia: a Geographical Information System Case Study. Geospatial Health. 2011;6: 77–84. 22109865 10.4081/gh.2011.159

[25] Nakhapakorn K, Tripathi NK. An Information Value Based Analysis of Physical and Climatic Factors Affecting Dengue Fever and Dengue Haemorrhagic Fever Incidence. Int J Health Geogr. 2005;4: 13 10.1186/1476-072X-4-13 15943863 PMC1177981

[26] Penna F, Lucia M. Ecological Study of Rio de Janeiro City DEN-3 Epidemic, 2001–2002. 2004; Available: http://repository.searo.who.int/handle/123456789/15955

[27] Reiter P, Lathrop S, Bunning M, Biggerstaff BJ, Singer D, Tiwari T, et al Texas Lifestyle Limits Transmission of Dengue Virus. Emerg Infect Dis. 2003;9: 86–89. 12533286 10.3201/eid0901.020220 PMC2873752

[28] Sarfraz MS, Tripathi NK, Tipdecho T, Thongbu T, Kerdthong P, Souris M. Analyzing the Spatio-temporal Relationship between Dengue Vector Larval Density and Land-use Using Factor Analysis and Spatial Ring Mapping. BMC Public Health. 2012;12: 853 10.1186/1471-2458-12-853 23043443 PMC3598814

[29] Seidahmed OME, Hassan SA, Soghaier MA, Siam HAM, Ahmed FTA, Elkarsany MM, et al Spatial and Temporal Patterns of Dengue Transmission along a Red Sea Coastline: A Longitudinal Entomological and Serological Survey in Port Sudan City. Kading RC, editor. PLoS Negl Trop Dis. 2012;6: e1821 10.1371/journal.pntd.0001821 23029582 PMC3459851

[30] Spiegel JM, Bonet M, Ibarra A-M, Pagliccia N, Ouellette V, Yassi A. Social and Environmental Determinants of *Aedes aegypti* Infestation in Central Havana: Results of a Case-control Study Nested in an Integrated Dengue Surveillance Programme in Cuba: Social and Environmental Determinants of *Aedes aegypti* Infestation. Trop Med Int Health. 2007;12: 503–510. 10.1111/j.1365-3156.2007.01818.x 17445141

[31] Stoddard ST, Forshey BM, Morrison AC, Paz-Soldan VA, Vazquez-Prokopec GM, Astete H, et al House-to-house Human Movement Drives Dengue Virus Transmission. Proc Natl Acad Sci. 2013;110: 994–999. 10.1073/pnas.1213349110 23277539 PMC3549073

[32] Thammapalo S, Chongsuwiwatwong V, Geater A, Lim A, Choomalee K. Socio-demographic and Environmental Factors Associated with *Aedes* Breeding Places in Phuket, Thailand. Southeast Asian J Trop Med Public Health. 2005;36: 426–433. 15916050

[33] Liebman KA, Stoddard ST, Morrison AC, Rocha C, Minnick S, Sihuincha M, et al Spatial Dimensions of Dengue Virus Transmission across Interepidemic and Epidemic Periods in Iquitos, Peru (1999–2003). Kent Crockett RJ, editor. PLoS Negl Trop Dis. 2012;6: e1472 10.1371/journal.pntd.0001472 22363822 PMC3283551

[34] Salje H, Lessler J, Endy TP, Curriero FC, Gibbons RV, Nisalak A, et al Revealing the Microscale Spatial Signature of Dengue Transmission and Immunity in an Urban Population. Proc Natl Acad Sci. 2012;109: 9535–9538. 10.1073/pnas.1120621109 22645364 PMC3386131

[35] Cummings DAT, Irizarry RA, Huang NE, Endy TP, Nisalak A, Ungchusak K, et al Travelling Waves in the Occurrence of Dengue Haemorrhagic Fever in Thailand. Nature. 2004;427: 344–347. 14737166 10.1038/nature02225

[36] Teurlai M, Huy R, Cazelles B, Duboz R, Baehr C, Vong S. Can Human Movements Explain Heterogeneous Propagation of Dengue Fever in Cambodia? PLoS Negl Trop Dis. 2012;6: e1957 10.1371/journal.pntd.0001957 23236536 PMC3516584

[37] Cuong HQ, Vu NT, Cazelles B, Boni MF, Thai KTD, Rabaa MA, et al Spatiotemporal Dynamics of Dengue Epidemics, Southern Vietnam. Emerg Infect Dis. 2013;19: 945–953. 10.3201/eid1906.121323 23735713 PMC3713821

[38] Hu W, Clements A, Williams G, Tong S, Mengersen K. Spatial Patterns and Socioecological Drivers of Dengue Fever Transmission in Queensland, Australia. Environ Health Perspect. 2011;120: 260–266. 10.1289/ehp.1003270 22015625 PMC3279430

[39] Lowe R, Bailey TC, Stephenson DB, Graham RJ, Coelho CAS, Sá Carvalho M, et al Spatio-temporal Modelling of Climate-sensitive Disease Risk: Towards an Early Warning System for Dengue in Brazil. Comput Geosci. 2011;37: 371–381. 10.1016/j.cageo.2010.01.008

[40] Wu P-C, Lay J-G, Guo H-R, Lin C-Y, Lung S-C, Su H-J. Higher Temperature and Urbanization Affect the Spatial Patterns of Dengue Fever Transmission in Subtropical Taiwan. Sci Total Environ. 2009;407: 2224–2233. 10.1016/j.scitotenv.2008.11.034 19157509

[41] Carbajo AE, Cardo MV, Vezzani D. Is Temperature the Main Cause of Dengue Rise in Non-endemic Countries? The Case of Argentina. Int J Health Geogr. 2012;11: 26 10.1186/1476-072X-11-26 22768874 PMC3517391

[42] Tipayamongkholgul M, Lisakulruk S. Socio-geographical Factors in Vulnerability to Dengue in Thai Villages: a Spatial Regression Analysis. Geospatial Health. 2011;5: 191–198. 21590669 10.4081/gh.2011.171

[43] Descloux E, Mangeas M, Menkes CE, Lengaigne M, Leroy A, Tehei T, et al Climate-Based Models for Understanding and Forecasting Dengue Epidemics. PLoS Negl Trop Dis. 2012;6: e1470 10.1371/journal.pntd.0001470 22348154 PMC3279338

[44] R Core Team. R: A Language and Environment for Statistical Computing. R Foundation for Statistical Computing, Vienna, Austria [Internet]. 2014 Available: http://www.R-project.org/

[45] Météo France. Formulaire de demande de données [Internet]. 2014 [cited 21 Jul 2014]. Available: http://www.meteo.nc/donnees-publiques-enseignement

[46] Li D, Liu W, Guigon A, Mostyn C, Grant R, Aaskov J. Rapid Displacement of Dengue Virus Type 1 by Type 4, Pacific Region, 2007–2009. Emerg Infect Dis. 2010;16: 123–125. 10.3201/eid1601.091275 20031057 PMC2874383

[47] Bouldouyre MA, Baumann F, Berlioz-Arthaud A, Chungue E, Lacassin F. Factors of Severity at Admission During an Epidemic of Dengue 1 in New Caledonia (South Pacific) in 2003. Scand J Infect Dis. 2006;38: 675–681. 10.1080/00365540600606432 16857614

[48] A-Nuegoonpipat A, Berlioz-Arthaud A, Chow V, Endy T, Lowry K, Mai LQ, et al Sustained Transmission of Dengue Virus Type 1 in the Pacific Due to Repeated Introductions of Different Asian Strains. Virology. 2004;329: 505–512. 10.1016/j.virol.2004.08.029 15518827

[49] Ahmad OB, Boschi-Pinto C, Lopez AD, Murray CJL, Lozano R, Inoue M. Age Standardisation of Rates: a New WHO Standard. World Health Organization; 2001 p. 14. Report No.: 31.

[50] Institut de la Statistique et des Etudes Economiques (ISEE). Recensement de la Population—1989. Institut de la Statistique et des Etudes Economiques; 1989.

[51] Institut de la Statistique et des Etudes Economiques (ISEE). Recensement de la Population—1996. Institut de la Statistique et des Etudes Economiques; 1996.

[52] Institut de la Statistique et des Etudes Economiques (ISEE). Recensement de la Population—2004. Institut de la Statistique et des Etudes Economiques; 2004.

[53] Institut de la Statistique et des Etudes Economiques (ISEE). Recensement de la Population—2009 [Internet]. 2009 [cited 21 Nov 2011]. Available: http://www.isee.nc/population/population.html

[54] IPNC. Rapport Technique—Année 1996. Nouméa: Institut Pasteur de Nouvelle-Calédonie; 1996.

[55] IPNC. Rapport Technique—Année 1999. Nouméa: Institut Pasteur de Nouvelle-Calédonie; 1999.

[56] IPNC. Rapport Technique—Année 2003 [Internet]. Nouméa: Institut Pasteur de Nouvelle-Calédonie; 2003 p. 154 Available: www.institutpasteur.nc/rapports-dactivites/

[57] Leroy A. Prévisibilité des Epidémies de Dengue par Arpège à Echéance Saisonnière—Prévisibilité des Fréquences d’Occurrence de Jours Chauds et Jours Pluvieux. Météo France; 2011 12 p. 27.

[58] Hijmans RJ, Cameron SE, Parra JL, Jones PG, Jarvis A. Very High Resolution Interpolated Climate Surfaces for Global Land Areas. Int J Climatol. 2005;25: 1965–1978. 10.1002/joc.1276

[59] Taylor KE, Stouffer RJ, Meehl GA. An Overview of CMIP5 and the Experiment Design. Bull Am Meteorol Soc. 2012;93: 485–498. 10.1175/BAMS-D-11-00094.1

[60] Anonymous. Program for Climate Model Diagnosis and Intercomparison [Internet]. [cited 25 Jul 2014]. Available: http://www-pcmdi.llnl.gov/

[61] Bellenger H, Guilyardi E, Leloup J, Lengaigne M, Vialard J. ENSO Representation in Climate Models: from CMIP3 to CMIP5. Clim Dyn. 2014;42: 1999–2018. 10.1007/s00382-013-1783-z

[62] Cavarero V, Peltier A, Aubail X, Leroy A, Dubuisson B, Jourdain S, et al Les Evolutions Passées et Futures du Climat de la Nouvelle-Calédonie. La Météorologie. 2012;77: 13–21.

[63] Bonvallot J, Gay J-C, Habert E. Atlas de la Nouvelle-Calédonie. Marseille: IRD Orstom; 2013.

[64] Bivand RS, Pebesma E, Gómez-Rubio V. Applied Spatial Data Analysis with R. 2nd ed. Springer New York; 2013.

[65] Escoffier B, Pagès J. Analyses Factorielles Simples et Multiples. Objectifs, Méthodes et Interprétation. 4th ed. Paris: Dunod; 2008.

[66] Dormann CF, Elith J, Bacher S, Buchmann C, Carl G, Carré G, et al Collinearity: a Review of Methods to Deal with It and a Simulation Study Evaluating their Performance. Ecography. 2013;36: 27–46. 10.1111/j.1600-0587.2012.07348.x

[67] Scherrer B. Biostatistique : Volume 1 2e édition Montréal: Gaëtan Morin; 2008.

[68] Vapnik V. Statistical Learning Theory. New York: Wiley; 1998.

[69] Cortes C, Vapnik V. Support-vector Networks. Mach Learn. 1995;20: 273–297.

[70] Ben-Hur A, Weston J. A User’s Guide to Support Vector Machines In: Carugo O, Eisenhaber F, editors. Data Mining Techniques for the Life Sciences. Totowa, NJ: Humana Press; 2010.

[71] Kohavi R. A Study of Cross-validation and Bootstrap for Accuracy Estimation and Model Selection. IJCAI 1995 pp. 1137–1145.

[72] Teurlai M. Modélisation Multi-échelle de la Dynamique Spatiale de la Dengue : Application à la Nouvelle-Calédonie et à la Région Pacifique. Université Montpellier II 2014.

[73] Couret J, Dotson E, Benedict MQ. Temperature, Larval Diet, and Density Effects on Development Rate and Survival of *Aedes aegypti* (Diptera: Culicidae). PLoS ONE. 2014;9: e87468 10.1371/journal.pone.0087468 24498328 PMC3911954

[74] Focks DA, Brenner RJ, Hayes J, Daniels E. Transmission Thresholds for Dengue in Terms of *Aedes aegypti* Pupae per Person with Discussion of Their Utility in Source Reduction Efforts. Am J Trop Med Hyg. 2000;62: 11–18. 10761719

[75] Mohammed A, Chadee DD. Effects of Different Temperature Regimens on the Development of *Aedes aegypti* (L.) (Diptera: Culicidae) Mosquitoes. Acta Trop. 2011;119: 38–43. 10.1016/j.actatropica.2011.04.004 21549680

[76] Dégallier N. Capacidade Vetorial : Importancia na Modelagem da Transmissao de Dengue In: Marcondes CB, editor. Entomologia Medica e Veterinaria : segunda ediçao. Sao Paulo: Atheneu; 2011 pp. 37–44.

[77] Watts DM, Burke DS, Harrison BA, Whitmire RE, Nisalak A. Effect of Temperature on the Vector Efficiency of *Aedes aegypti* for Dengue 2 Virus. Am J Trop Med Hyg. 1987;36(1): 143–152. 3812879 10.4269/ajtmh.1987.36.143

[78] Hopp MJ, Foley JA. Global-scale Relationships between Climate and the Dengue Fever Vector, *Aedes aegypti* . Clim Change. 2001;48: 441–463.

[79] Yang HM, Macoris MLG, Galvani KC, Andrighetti MTM, Wanderley DMV. Assessing the Effects of Temperature on the Population of *Aedes aegypti*, the Vector of Dengue. Epidemiol Infect. 2009;137: 1188–1202. 10.1017/S0950268809002040 19192322

[80] Scott TW, Amerasinghe PH, Morrison AC, Lorenz LH, Clark GG, Strickman D, et al Longitudinal Studies of *Aedes aegypti* (Diptera: Culicidae) in Thailand and Puerto Rico: Blood Feeding Frequency. J Med Ent. 2000;37: 89–101. 10.1603/0022-2585-37.1.89 15218911

[81] Brady OJ, Johansson MA, Guerra CA, Bhatt S, Golding N, Pigott DM, et al Modelling Adult *Aedes aegypti* and *Aedes albopictus* Survival at Different Temperatures in Laboratory and Field Settings. Parasit Vectors. 2013;6: 351 10.1186/1756-3305-6-351 24330720 PMC3867219

[82] Stewart Ibarra AM, Ryan SJ, Beltrán E, Mejía R, Silva M, Muñoz Á. Dengue Vector Dynamics (*Aedes aegypti*) Influenced by Climate and Social Factors in Ecuador: Implications for Targeted Control. Mores CN, editor. PLoS ONE. 2013;8: e78263 10.1371/journal.pone.0078263 24324542 PMC3855798

[83] Degallier N, Vilarinhos P d. TR, Carvalho M d. SL d., Knox MB, Caetano J. People’s Knowledge and Practice about Dengue, Its Vectors and Control Means in Brasilia (DF), Brazil: Its Relevance with Entomological Factors. J Amer Mosq Cont Assoc. 2000;16 (2): 114–123. 10901634

[84] Getis A, Morrison AC, Gray K, Scott TW. Characteristics of the Spatial Pattern of the Dengue Vector, *Aedes aegypti*, in Iquitos, Peru. Am J Trop Med Hyg. 2003;69: 494–505. 14695086

[85] Harrington LC, Scott TW, Lerdthusnee K, Coleman RC, Costero A, Clark GG, et al Dispersal of the Dengue Vector *Aedes aegypti* within and between Rural Communities. AmJ.tropMedHyg. 2005;72: 209–220. 15741559

[86] Maciel-de-Freitas R, Codeco CT, Lourenço-de-Oliveira R. Body Size-associated Survival and Dispersal Rates of *Aedes aegypti* in Rio de Janeiro. Med Vet Entomol. 2007;21: 284–292. 17897370 10.1111/j.1365-2915.2007.00694.x

[87] Muir LE, Kay BH. *Aedes aegypti* Survival and Dispersal Estimated by Mark-release-recapture in Northern Australia. Am J Trop Med Hyg. 1998;58 (3): 277–282. 9546403 10.4269/ajtmh.1998.58.277

[88] Aldstadt J, Yoon I-K, Tannitisupawong D, Jarman RG, Thomas SJ, Gibbons RV, et al Space-time Analysis of Hospitalised Dengue Patients in Rural Thailand Reveals Important Temporal Intervals in the Pattern of Dengue Virus Transmission: Space-time Dengue Transmission Intervals. Trop Med Int Health. 2012;17: 1076–1085. 10.1111/j.1365-3156.2012.03040.x 22808917 PMC4099473

[89] Mammen MP Jr, Pimgate C, Koenraadt CJ, Rothman AL, Aldstadt J, Nisalak A, et al Spatial and Temporal Clustering of Dengue Virus Transmission in Thai Villages. PLoS Med. 2008;5: e205 10.1371/journal.pmed.0050205 18986209 PMC2577695

[90] Morrison AC, Getis A, Santigo M, Rigau-Perez JG, Reiter P. Exploratory Space-time Analysis of Reported Dengue Cases During an Outbreak in Florida, Puerto Rico, 1991–1992. Am J Trop Med Hyg. 1998;58: 287–298. 9546405 10.4269/ajtmh.1998.58.287

[91] Rabaa MA, Klungthong C, Yoon I-K, Holmes EC, Chinnawirotpisan P, Thaisomboonsuk B, et al Frequent In-Migration and Highly Focal Transmission of Dengue Viruses among Children in Kamphaeng Phet, Thailand. Harris E, editor. PLoS Negl Trop Dis. 2013;7: e1990 10.1371/journal.pntd.0001990 23350000 PMC3547850

[92] Tran A, Deparis X, Dussart P, Morvan J, Rabarisn P, Remy F, et al Dengue Spatial and Temporal Patterns, French Guiana, 2001. Emerg Infect Dis. 2004;10: 615–621. 15200850 10.3201/eid1004.030186 PMC3323097

[93] Vazquez-Prokopec GM, Kitron U, Montgomery B, Horne P, Ritchie SA. Quantifying the Spatial Dimension of Dengue Virus Epidemic Spread within a Tropical Urban Environment. PLoS Negl Trop Dis. 2010;4: e920 10.1371/journal.pntd.0000920 21200419 PMC3006131

[94] Yoon I-K, Getis A, Aldstadt J, Rothman AL, Tannitisupawong D, Koenraadt CJM, et al Fine Scale Spatiotemporal Clustering of Dengue Virus Transmission in Children and *Aedes aegypti* in Rural Thai Villages. PLoS Negl Trop Dis. 2012;6: e1730 10.1371/journal.pntd.0001730 22816001 PMC3398976

[95] Dufour B, Hendrikx P. Surveillance Epidémiologique en Santé Animale. 2ème ed. Cirad; 2005.

[96] Endy TP, Chunsuttiwat S, Nisalak A, Libraty DH, Green S, Rothman AL, et al Epidemiology of Inapparent and Symptomatic Acute Dengue Virus Infection: A Prospective Study of Primary School Children in Kamphaeng Phet, Thailand. Am J Epidemiol. 2002;156: 40–51. 10.1093/aje/kwf005 12076887

[97] Endy TP, Anderson KB, Nisalak A, Yoon I-K, Green S, Rothman AL, et al Determinants of Inapparent and Symptomatic Dengue Infection in a Prospective Study of Primary School Children in Kamphaeng Phet, Thailand. PLoS Negl Trop Dis. 2011;5: e975 10.1371/journal.pntd.0000975 21390158 PMC3046956

[98] Dormann CF, M. McPherson J, B. Araújo M, Bivand R, Bolliger J, Carl G, et al Methods to Account for Spatial Autocorrelation in the Analysis of Species Distributional Data: a Review. Ecography. 2007;30: 609–628. 10.1111/j.2007.0906–7590.05171.x

[99] Dupont-Rouzeyrol M, Caro V, Guillaumot L, Vazeille M, D’Ortenzio E, Thiberge J-M, et al Chikungunya Virus and the Mosquito Vector *Aedes aegypti* in New Caledonia (South Pacific Region). Vector Borne Zoonotic Dis Larchmt N. 2012;12: 1036–1041. 10.1089/vbz.2011.0937 23167500

