# Supplementary figures and images for: Socio-economic and Climate Factors Associated with Dengue Fever Spatial Heterogeneity: A Worked Example in New Caledonia
Source: PLoS Negl Trop Dis. 2015 Dec 1;9(12):e0004211. doi: 10.1371/journal.pntd.0004211 (PMC4666598; doi:10.1371/journal.pntd.0004211)

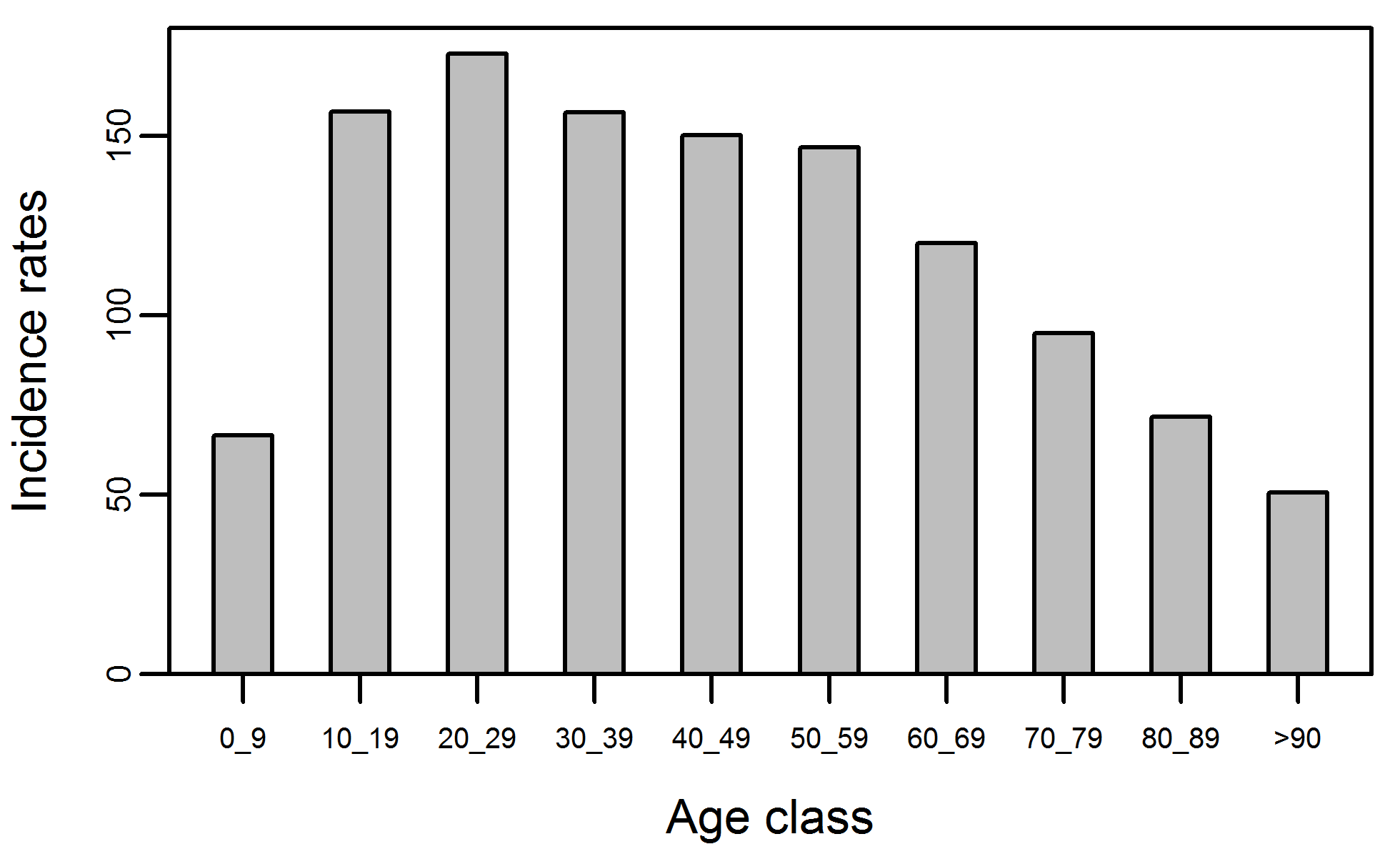

Supplement: S1 Fig — Incidence rates are averaged over epidemic years between 1995 and 2012 and are shown in number of cases per 10,000 people per year. (TIF) [file pntd.0004211.s001.tif]

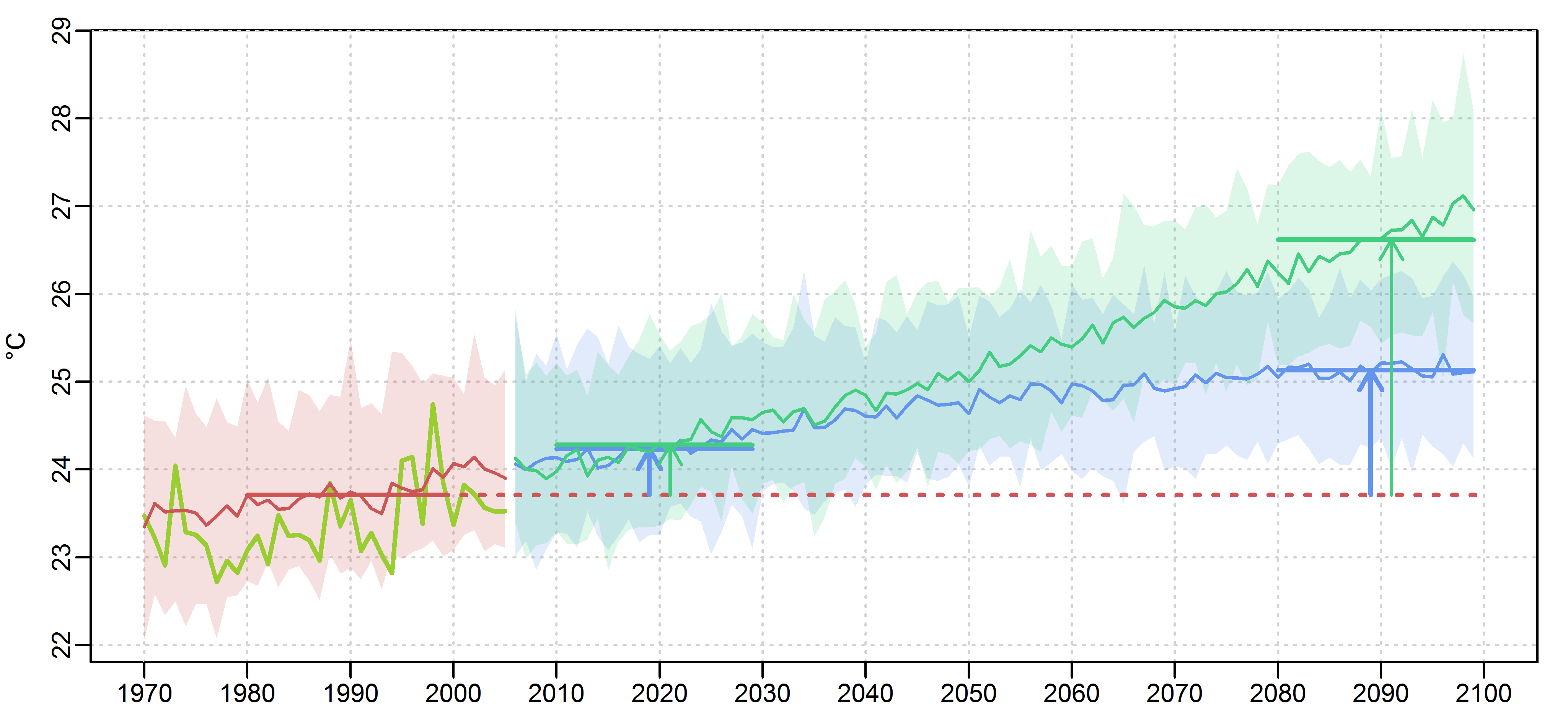

Supplement: S2 Fig — The yellow-green curve (1971–2005) represents the observed mean temperature in Noumea. The other curves represent the average of the annual time series simulated by the ten models over the historical period (1971–2005, red curve), and the 2006–2099 period under RCP 4.5 (blue curve) and RCP 8.5 (green curve) scenarios. Horizontal segments represent the average of the time series over the given periods (1980–1999, 2010–2029 and 2080–2099). Arrows represent the average increase in mean temperature for each time period (see Table 3). Background shadings represent the annual range of mean temperature simulations over the ten models. (TIF) [file pntd.0004211.s002.tif]

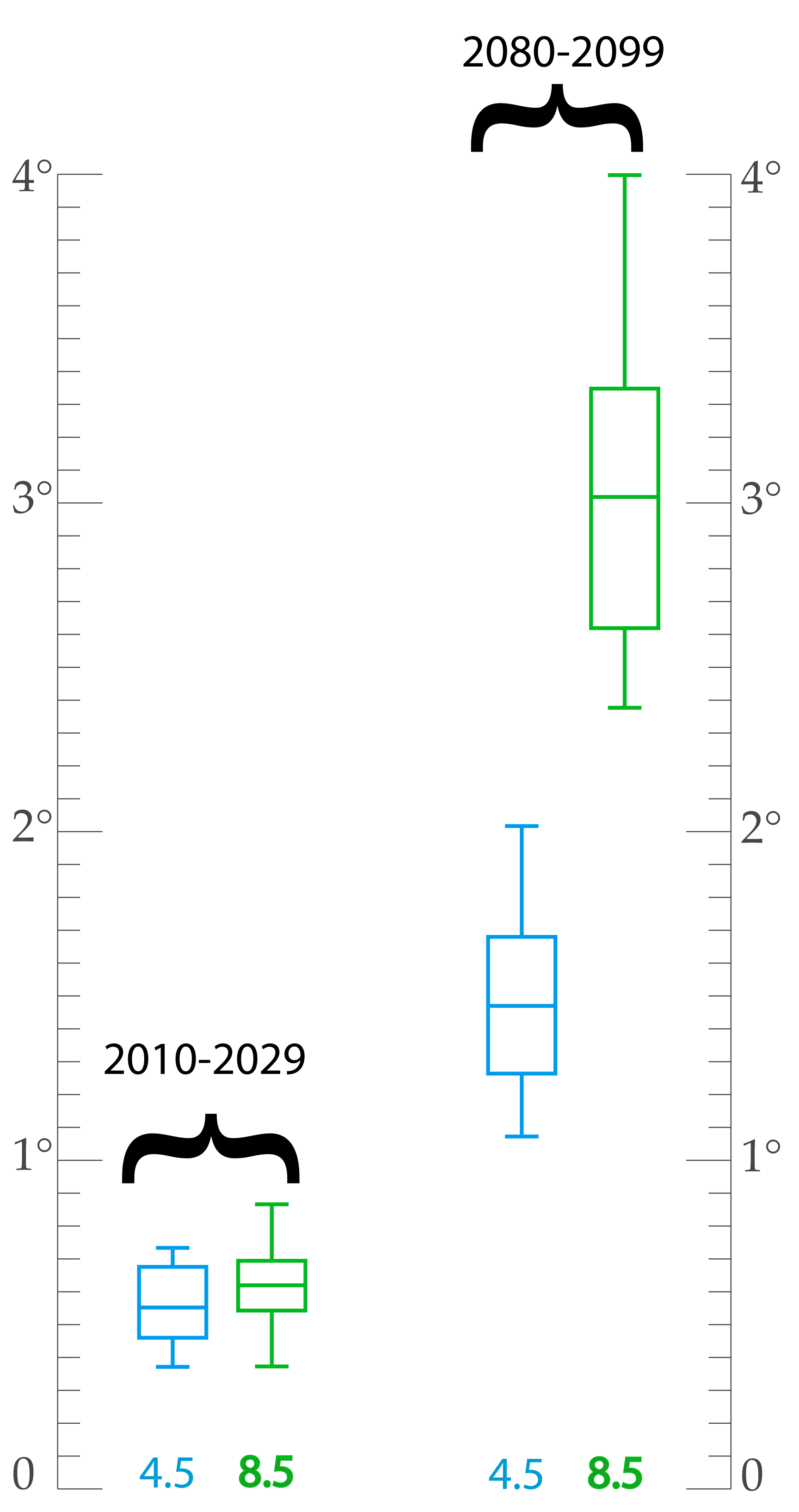

Supplement: S3 Fig — The boxplot (over the ten GCM selected) of the average increase in mean temperature is given for two climate change scenarios (RCP 4.5 and RCP 8.5) and two time periods relative to the historical series of temperature (1980–1999). (TIFF) [file pntd.0004211.s003.tiff]

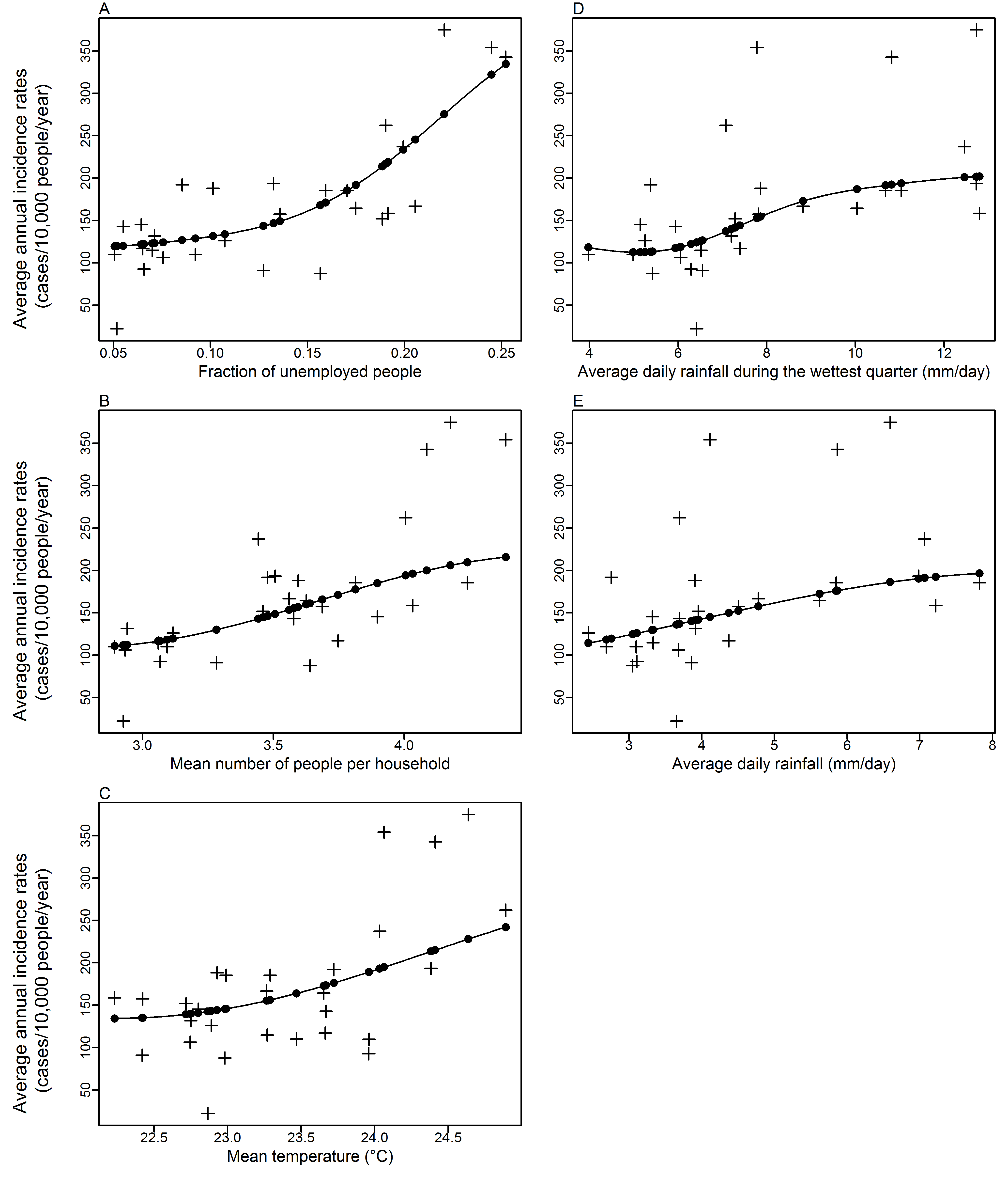

Supplement: S4 Fig — For each selected explanatory variable (A to E), mean annual incidence rates observed in 28 communes of New Caledonia (crosses), and mean annual incidence rates predicted by the univariable SVM model based on the corresponding explanatory variable (dots). The curve represents the univariable SVM model predictions over the whole observed range of the explanatory variable (non-linear regression curve). RMSE of each model are 53 (A), 68 (B), 69 (C), 72 (D), 75 (E), as presented in Table 2. (TIFF) [file pntd.0004211.s004.tiff]

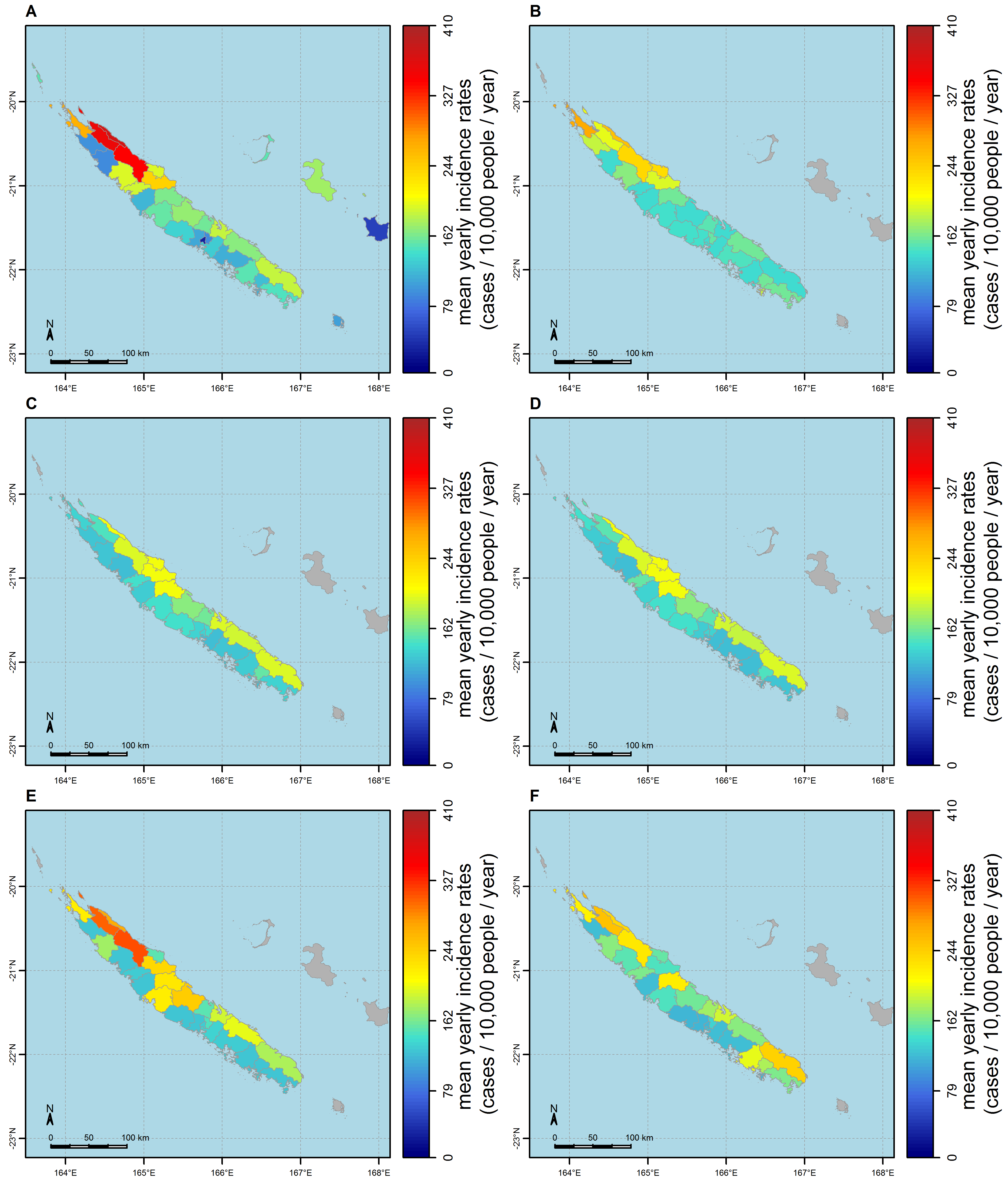

Supplement: S5 Fig — A: map of the observed mean dengue annual incidence rates. B to E: maps of mean annual incidence rates predicted by univariable SVM models based only on one of the 5 selected variables: mean temperature (B), average daily rainfall (C), average daily rainfall during the wettest quarter (D), percentage of unemployed people (E) and mean number of people per household (F). (TIF) [file pntd.0004211.s005.tif]
